# Proton–electron temporal asynchrony on femtosecond timescales enables anti-corrosive low-iridium anodes for PEM electrolysers

**DOI:** 10.1038/s41565-026-02136-x

**Published:** 2026-02-27

**Authors:** Wei Shen, Fei-Yue Gao, Xiaogang Sun, Haodian Xie, Yang Hu, Huiying Wu, Mietek Jaroniec, Yao Zheng, Pinxian Xi, Chun-Hua Yan, Shi-Zhang Qiao

**Affiliations:** 1 State Key Laboratory of Natural Product Chemistry, Frontiers Science Center for Rare Isotopes, College of Chemistry and Chemical Engineering, Lanzhou University, Lanzhou, China; 2 School of Chemical Engineering, The University of Adelaide, Adelaide, South Australia Australia; 3 Department of Chemistry and Biochemistry and Advanced Materials and Liquid Crystal Institute, Kent State University, Kent, OH USA; 4 State Key Laboratory of Baryunobo Rare Earth Resource Research and Comprehensive Utilization, Baotou Research Institute of Rare Earths, Baotou, China; 5 Beijing National Laboratory for Molecular Sciences, State Key Laboratory of Rare Earth Materials Chemistry and Applications, PKU-HKU Joint Laboratory in Rare Earth Materials and Bioinorganic Chemistry, College of Chemistry and Molecular Engineering, Peking University, Beijing, China

**Keywords:** Electrocatalysis, Electrocatalysis

## Abstract

The development of corrosion-resistant low-iridium anode catalysts is the key challenge in proton exchange membrane water electrolysis. However, the fundamental origin of anodic corrosion has been intensely debated over the years, mainly because of the limited mechanistic understanding of the complex proton-coupled electron transfer process. In this work, we employed femtosecond electrochemical transient absorption spectroscopy to probe the spatial–temporal synchronization of protons and electrons during the elementary proton-coupled electron transfer step at the femtosecond (10^−15^ s) timescale. Here we show that anodic corrosion is initiated within 100 fs after polarization startup, driven by synchronized protons and electrons coupling at the electrode surface. By introducing a Lewis acid (CeO_2_) as a proton channel, the reaction dynamics of protons and electrons could be decoupled into temporal asynchrony to prevent the generation of soluble Ir^6+^ species. Owing to this unique desynchronized proton–electron interaction, the CeO_2_–IrO_2_ catalyst demonstrates outstanding stability for about 1,400 h of continuous operation.

## Abstract (web summary)

Femtosecond electrochemical transient absorption spectroscopy is employed to explore the fundamental origin of anodic corrosion. It reveals that Ir corrosion originates from ultrafast proton–electron synchronization and that CeO_2_ can create femtosecond-scale proton–electron asynchrony for corrosion-resistant low-Ir anodes.

## Main

Proton exchange membrane water electrolysis (PEMWE) is an ideal technology for sustainable hydrogen production using renewable energy^1,2^. However, the high cost of the catalyst hampers its large-scale application, necessitating the development of anode catalysts with reduced precious iridium (Ir) loading. However, at present, the corrosion of low-content Ir in PEMWE urgently needs to be solved, but the fundamental nature of the evolution of corrosion remains under intensive debate^3,4^. Previous studies have shown that the formation of highly active Ir^6+^ species is a major cause of corrosion owing to its dissolution^3^. Therefore, the developed protection mechanisms mainly focus on steady-state material engineering strategies, such as accelerating charge transfer, applying armour protection and passivation layers, and so on^5–7^. These works have elucidated the reaction mechanisms and proposed catalyst design from an energetic perspective.

However, in the electrocatalysis of oxygen evolution reactions (OER), the transient mechanisms involving reaction intermediates and protons/electrons evolving over time have not yet been explored^8,9^. The anodic transformations triggered by start–stop events are typically transient and elusive, unlike steady-state operation, posing the challenges for direct observation^10–12^. In contrast to the well-studied evolution of stable intermediates such as *OH, *O and *OOH in the OER, the spatiotemporal evolution of critical Ir^6^⁺ species in transient proton-coupled electron transfer (PCET) steps remains poorly understood^13,14^. In particular, the microscopic kinetics of proton and electron evolution in PCET has evaded clear probing and precise control. The absence of microscopic insights regarding the initial state makes the prediction of the subsequent evolution of the macroscopic material structure challenging, thereby hindering the establishment of effective anti-corrosive design principles^15,16^.

Currently, the identification of steady-state catalytic active sites mainly relies on spectroscopic techniques with temporal resolution on the millisecond (10^−3^ s) to microsecond (10^−6^ s) level, such as electron energy loss spectroscopy, Fourier transform infrared spectroscopy and X-ray absorption spectroscopy^17–25^. However, the elementary steps of chemical bond breaking/formation in PCET are essentially ultrafast processes at the picosecond (10^−12^ s) to femtosecond (10^−15^ s) level. Owing to this timescale difference of six or more orders of magnitude between the elementary reactions and the resolution of current spectroscopic techniques, existing analytical methods face challenges in capturing these ultrafast processes to understand the catalytic mechanism^26–30^. The lack of cross-scale correlations between macroscopic steady-state characterization and microscopic transient dynamics prevents existing theories from resolving the spatiotemporal evolution sequence of active sites and reaction intermediates. This gap in the temporal dimension severely restricts the manipulation of catalytic reactions owing to ultrafast time-resolved perspectives.

In this Article, we used femtosecond electrochemical transient absorption spectroscopy (fs-ECTAS) to identify and decouple the temporal sequence steps in multistep PCET reactions on a typical IrO_2_ anode. By bridging isotope labelled macroscopic proton transport kinetics with femtosecond-resolved microscopic dynamics, we determined that the corrosion of commercial IrO_2_ originates from the synchronous presence of protons and electrons before 100 fs. On the basis of this, a Lewis acid, CeO_2_, which acts as a proton transfer channel, was introduced to regulate the temporal asynchrony between proton and electron transfer. Such modulation enabled electron transfer within the first 100 fs, while proton transfer from water oxidation occurred subsequently, within the 100–300 fs timescale. This spatiotemporal asynchrony enhanced the kinetics of proton transfer and stabilized transient Ir^6+^ species for improved anode corrosion resistance. This work represents an investigation of electrocatalytic processes and mechanisms at the femtosecond timescale, offering a new perspective to overcome theoretical and mechanistic bottlenecks in electrocatalysis.

## Origin of commercial IrO_2_ corrosion

**Fig. 1:**
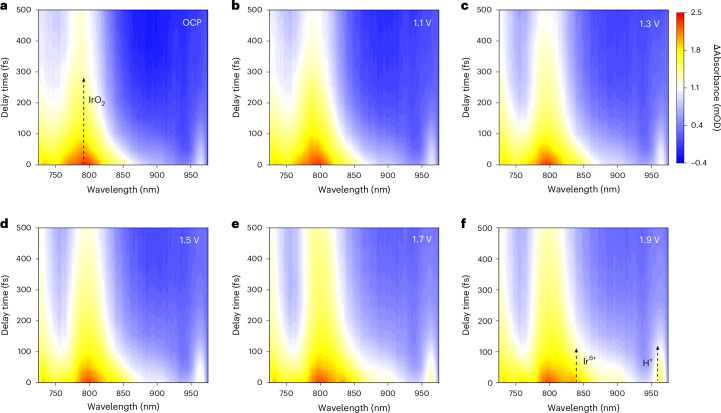
Corrosion issues of commercial IrO_2_. **a–f**, fs-ECTAS of IrO_2_ under OCP (**a**) and 1.1 V (**b**), 1.3 V (**c**), 1.5 V (**d**), 1.7 V (**e**) and 1.9 V (**f**) potentials in 0.5 M H_2_SO_4_. Arrows indicate the characteristic signal peaks assigned to IrO_2_, Ir^6+^ and H^+^species, respectively. Milli-optical density (mOD) denotes the magnitude of the differential absorbance intensity. Source data

We report the development of fs-ECTAS that merged femtosecond pump–probe spectroscopy with an in situ electrochemical platform (Supplementary Fig. 1). During measurement, the pump beam acted as a photonic internal standard to precisely define the reaction time zero. Under the influence of external voltage, the selective excitation by pump photons enabled the labelling of distinct electronic states within the catalyst. These electronic states were influenced by interfacial charge transfer processes driven by the external field, giving rise to transient optical features associated with chemical bond reorganization on the femtosecond to picosecond timescale. We then captured the interfacial proton–electron evolution dynamics on a commercial IrO_2_ catalyst under working potentials (Fig. 1a–f and Supplementary Figs. 2–4). As shown in Fig. 1a, the excited-state absorption of IrO_2_ (790 nm) can all be observed under the steady-state condition of open circuit potential (OCP). With increasing voltage, two absorption bands attributed to Ir^6+^ species and H^+^ in the hydrogen bond network emerged at 830 nm and 963 nm, respectively (Fig. 1b–f). The Ir^6+^ species was identified on the basis of the correlation between the oxidation process and the potential-dependent absorption evolution observed in the ECTAS spectra, which was further confirmed by density functional theory (DFT) calculations to simulate their excited-state energy (Supplementary Fig. 5). The calculated excitation energy differences correspond to the characteristic absorption wavelengths observed in the transient absorption spectra. The results showed that the excited-state absorption peaks of IrO_2_, Ir^6+^ and H^+^ were located at 782.6 nm, 838.8 nm and 971.2 nm, respectively. The formation of Ir^6+^ at a high potential (1.9 V) and the adsorption of surface protons occurred simultaneously within 100 fs, suggesting that this was an electron–proton coupling process at the sub-picosecond timescale.

We conducted DFT to reveal the influence of protons and electrons on the dissolution behaviour of Ir (Supplementary Figs. 6–8). As shown in Supplementary Fig. 9, the calculated cationic vacancy formation energy was 7.75 eV when only electron accumulation occurred at the Ir site, and 5.37 eV when only proton adsorption was considered. In contrast, the formation energy decreased to 5.26 eV when both proton and electron effects were present. These results indicate that the simultaneous presence of protons and electrons lowered the cationic vacancy formation energy, thereby promoting the dissolution corrosion of Ir. Proton transfer typically lags behind electron transfer, resulting in proton accumulation. This undesirable proton–electron synchronization is the key factor for Ir corrosion. Therefore, it was essential to accelerate proton transport and prevent its accumulation to mitigate Ir corrosion during OER.

## Promotion of proton transport by CeO_2_–IrO_2_

**Fig. 2:**
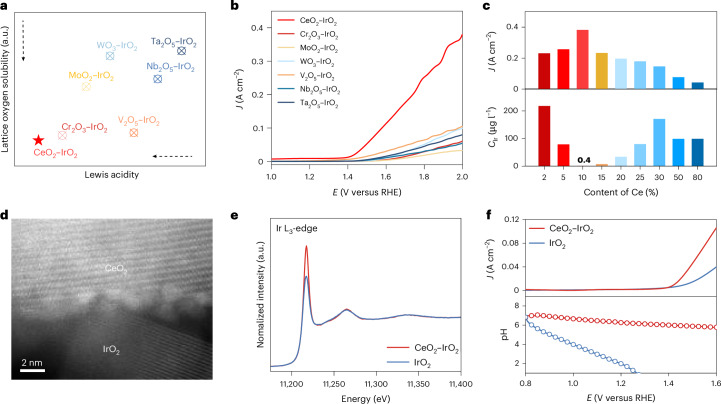
Electrochemical properties of CeO_2_–IrO_2_. **a**,**b**, The lattice oxygen solubility (**a**) and LSV (**b**) of MO–IrO_2_ (where M is Ce, Ta, Nb, V, W, Mo or Cr). **c**, A performance and Ir dissolution comparison of CeO_2_–IrO_2_ with different Ce ratios. *J* denotes the current density, *E* represents the applied potential and *C* indicates the concentration. **d**,**e**, A high angle annular dark field scanning transmission electron microscope image (**d**) and X-ray absorption spectroscopy plot (**e**) of CeO_2_–IrO_2_. **f**, The surface pH (bottom) and *J* (top) of CeO_2_–IrO_2_ and IrO_2_ detected by rotating ring disk electrode. Source data

On the basis of the results of the fs-ECTAS characterization and DFT computation, we synthesized a series of Lewis acidic metal oxide and IrO_2_ composite materials (MO–IrO_2_, where M is Ce, Ta, Nb, V, W, Mo or Cr) to accelerate proton transfer. As shown in Fig. 2a and Supplementary Figs. 10 and 11, the relatively weak Lewis acidity and low lattice oxygen solubility of Ce provided a soft proton conduction channel, facilitating rapid proton migration. Performance evaluation revealed that CeO_2_–IrO_2_ with 10% CeO_2_ exhibited the best catalytic performance (Fig. 2b,c and Supplementary Fig. 12). The physicochemical characterization of CeO_2_–IrO_2_ confirmed that CeO_2_ was supported on IrO_2_ (Fig. 2d,e and Supplementary Figs. 13–18). This revealed that the enhanced catalytic performance of the system was probably because of the rapid proton transfer across the CeO_2_–IrO_2_ interface without accumulation, rather than the conventional modulation of the electronic structure at metal active sites (Fig. 2f and Supplementary Figs. 19 and 20).

As a result, the CeO_2_–IrO_2_ catalyst exhibited no noticeable degradation under the accelerated corrosion tests with frequent start–stop cycling, while commercial IrO_2_ underwent severe degradation after approximately 1,500 cycles, leading to a rapid increase in cell voltage (Supplementary Fig. 21). This enhanced anti-corrosive ability of CeO_2_–IrO_2_ was further confirmed by the inductively coupled plasma measurements of Ir dissolution under constant current electrolysis (Supplementary Fig. 22). These findings indicate that CeO_2_–IrO_2_ underwent much less Ir dissolution, indicating that CeO_2_ effectively suppresses Ir corrosion and leaching during operation, which was further supported by more in situ and ex situ structural characterizations (Supplementary Figs. 23–25).

**Fig. 3:**
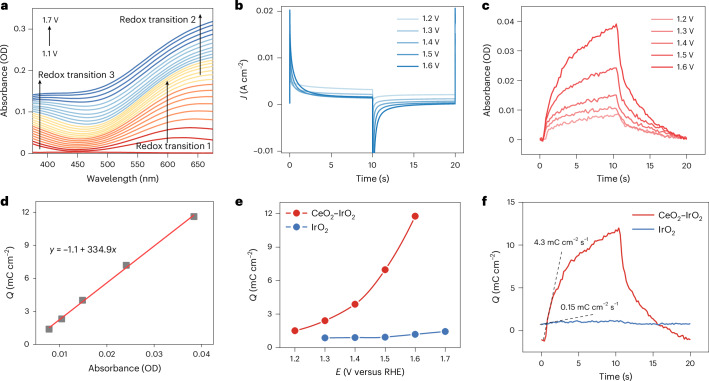
Evidence of electron transfer. **a**, In situ UV–vis absorption spectra of CeO_2_–IrO_2_ with Ir species in three sequential redox transitions^15^. **b**–**d**, The pulse–current (**b**), pulse–absorbance (**c**) and charge–absorption (**d**) relationships in time-resolved UV–vis absorption spectra of CeO_2_–IrO_2_. **e**, The surface charge density as a function of potential for CeO_2_–IrO_2_ and IrO_2_. **f**, The evolution of the surface charge density with the pulse duration for CeO_2_–IrO_2_ and IrO_2_. Source data

The in situ electrochemical ultraviolet–visible (UV–vis) absorption technique was applied to detect Ir oxidation states under various potentials^31^. The differential features observed in the UV–vis absorption spectra primarily reflected surface-related absorption changes. On the basis of these signals, the changes in catalyst valence states were precisely monitored^32,33^. In addition, it showed a linear relationship between the absorption intensity in UV–vis spectra and the number of oxidation state species. Thus, the charge-dependent bleaching behaviour of the spectra enabled accurate quantification of spectral changes induced by transient oxidation states^15,34,35^. As shown in Fig. 3a and Supplementary Fig. 26, potential-dependent UV–vis absorption spectroscopy revealed that the CeO_2_–IrO_2_ system exhibited stronger absorption characteristics of oxidation intermediates than the IrO_2_ system. Time-resolved UV–vis spectroscopy tracked the absorption changes of specific oxidation states under pulsed potentials. When the potential triggered an oxidation state change, the electronic structure shifted and altered the transition behaviour (Fig. 3b and Supplementary Fig. 27). As a result, the absorption increased when the potential rose, and returned to its initial value when the potential dropped (Fig. 3c). The adsorption signals were correlated with the extracted charge to calculate the molar extinction coefficients of the active species (Fig. 3d). The results showed that the Ir^6+^ active charge density of CeO_2_–IrO_2_ was an order of magnitude higher than that of the original IrO_2_ under the stepped potential of 1.6 V versus reversible hydrogen electrode (RHE) (Fig. 3e). This increased accumulation of active surface charges indicates that CeO_2_ plays a critical role in mitigating surface charge dissipation, which was otherwise induced by the dissolution of Ir^6+^ species. A charge transfer rate model was further constructed, enabling the extraction of time-dependent surface charge accumulation upon pulsed potential excitation (Fig. 3f). We then determined the electron transfer rate by differentiating Ir charge accumulation with respect to time. The charge transfer rate of CeO_2_–IrO_2_ (4.3 mC cm^−2^ s^−1^) was 28 times higher than that of commercial IrO_2_ (0.15 mC cm^−2^ s^−1^), demonstrating that Ce incorporation during catalysis stabilized the active Ir^6+^ species, thereby mitigating non-catalytic electron dissipation and suppressing corrosion-related side reactions.

## Kinetics of proton transfer

To further elucidate the relationship between the oxidation state transition of Ir^6+^ and its proton transfer behaviour, millisecond-level (10^−3^ s) time-resolved electrochemical quartz crystal microbalance measurements under pulsed potential control were performed. We further correlated the obtained charge with the differential mass change. As shown in Supplementary Fig. 28, integration of the current–time response at the end of the pulse revealed that the charge-to-mass (d*M*/*Q*) response coefficient of CeO_2_–IrO_2_ was higher than that of IrO_2_. This indicated that CeO_2_–IrO_2_ induced a more pronounced mass change associated with proton transfer for the same charge transfer. We then employed deuterium isotope tracing to investigate how CeO_2_ accelerates proton transfer, which was enabled by a customized membrane electrode assembly (MEA) cell integrated with differential electrochemical mass spectrometry. As shown in Supplementary Fig. 29, potential-dependent measurements revealed a marked increase in the D_2_/H_2_ ratio for the CeO_2_–IrO_2_ catalyst, reaching 2.05% at 1.8 V, which was 5.4 times higher than that of IrO_2_ (0.38%) under identical conditions. This suggests that CeO_2_ substantially lowered the activation energy barrier for proton transfer, thereby facilitating the efficient transport of heavier D^+^ ions across the membrane–electrode interface.

## Time-decoupled proton kinetics

**Fig. 4:**
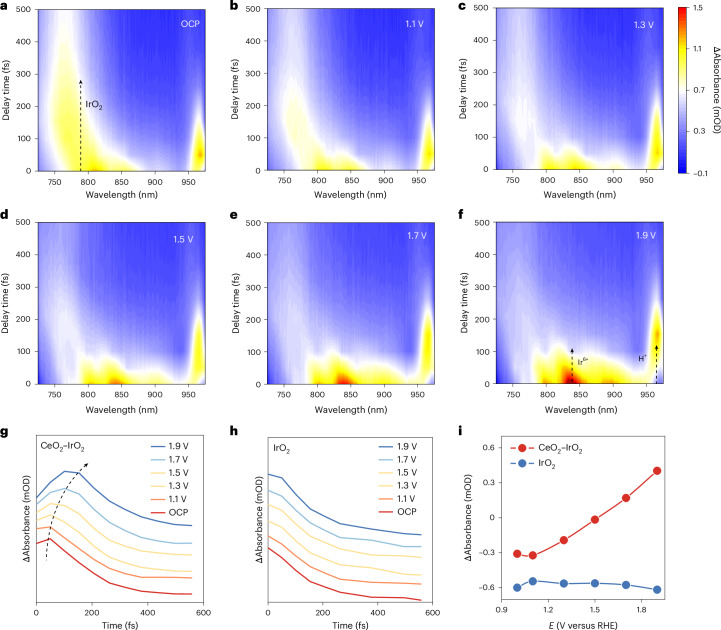
Proton transfer kinetics. **a–f**, fs-ECTAS of CeO_2_–IrO_2_ under OCP (**a**) and 1.1 V (**b**), 1.3 V (**c**), 1.5 V (**d**), 1.7 V (**e**) and 1.9 V (**f**) potentials in 0.5 M H_2_SO_4_. Arrows indicate the characteristic signal peaks assigned to IrO_2_, Ir^6+^ and H^+^ species, respectively. **g**,**h**, Transient absorption spectra of CeO_2_–IrO_2_ (**g**) and IrO_2_ (**h**) with proton dynamics curves. **i**, The microscopic proton dynamics of CeO_2_–IrO_2_ and IrO_2_. Source data

We revealed time-decoupled kinetic behaviour and its connection to the kinetics of proton evolution in the CeO_2_–IrO_2_ composite catalyst by fs-ECTAS (Fig. 4a–f). On the basis of the femtosecond timescale kinetic profile of proton evolution, we successfully established a quantitative correlation model between microscopic and macroscopic proton transfer kinetics. Compared with IrO_2_, CeO_2_–IrO_2_ exhibited a pronounced shift of the H^+^ kinetic peak towards a longer timescale during polarization (Fig. 4g,h). This indicated that the introduction of CeO_2_ effectively promoted the kinetics of proton transfer, thereby preventing the accumulation of protons. By extracting the differential absorption signals at 150 fs, we constructed a voltage-dependent proton transfer kinetic profile (Fig. 4i). The data revealed that the CeO_2_–IrO_2_ system displayed a higher sensitivity of differential absorption to potential variation compared with IrO_2_, confirming its superior proton transfer dynamics.

**Fig. 5:**
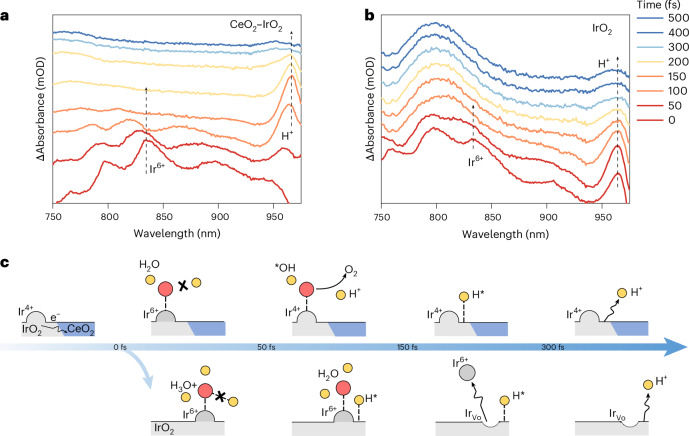
Ultrafast reaction mechanism of CeO_2_–IrO_2_. **a**, An fs-ECTAS of CeO_2_–IrO_2_ at 1.9 V versus RHE. **b**, An fs-ECTAS of IrO_2_ at 1.9 V versus RHE. Arrows indicate the characteristic signal peaks assigned to IrO_2_, Ir^6+^ and H^+^ species, respectively. **c**, A schematic illustration of the time-resolved catalytic mechanism for CeO_2_–IrO_2_ and IrO_2_. Ir_Vo_ denotes the lattice vacancy formed after Ir dissolution. Source data

On the basis of the above analysis, leveraging fs-ECTAS to decouple the kinetic features of key multistep anodic processes (proton and electron transfer) was crucial for elucidating the corrosion resistance mechanism of CeO_2_–IrO_2_ and guiding its rational optimization. As revealed by the time–wavelength–absorbance fs-ECTAS maps in Fig. 5a,b, the CeO_2_–IrO_2_ system exhibited distinctly different kinetics compared with IrO_2_. The CeO_2_–IrO_2_ system exhibited distinct charge transfer kinetics under OER conditions at 1.9 V versus RHE. Time-resolved absorption spectroscopy revealed that the Ir^6+^ absorption feature vanished within 100 fs, whereas the corresponding proton signal emerged at 100–300 fs. Kinetic analysis of the correlated absorbance signals within 500 fs further revealed that the incorporation of CeO_2_ established a spatiotemporal asynchronous mechanism. This temporal decoupling effectively disrupted the synchronization between Ir^6+^ and protons, thereby stabilizing Ir^6+^. This was attributed to the incorporation of CeO_2_, which introduced surface hydroxyl groups with strong oxophilicity, creating a low-energy pathway for proton transfer in the post electron transfer regime (100–300 fs). This temporal decoupling of proton–electron transfer markedly suppresses interfacial charge recombination and provides experimental insight into the dynamic regulation of PCET in anodic OER.

Time-resolved precise spectral analyses uncover a fundamental difference in proton transfer kinetics. In the CeO_2_–IrO_2_ system, proton-related signals emerged within 100 fs and decayed within 300 fs, indicating accelerated kinetics. This stands in sharp contrast to the longer proton characteristic duration (>500 fs) observed in the IrO_2_ system. This kinetic enhancement correlated directly with a distinct proton generation pathway. fs-ECTAS captured within 150 fs revealed the synchronous formation of Ir^6+^ intermediates from CeO_2_–IrO_2_, with their concurrent decay strongly correlated with the rise of the H^+^ signal after 100 fs. These findings indicate that protons in the CeO_2_–IrO_2_ system originated from the in situ oxidation of initial adsorption water (<50 fs) during OER, rather than from the residual protons accumulated in the previous OER cycle. As shown in Fig. 5a, the absorption peaks shifted markedly with time except for the H^+^ signal. This behaviour was closely associated with electron transfer at the Ir active centres and the evolution of surface-adsorbed species during the reaction. Voltage-dependent measurements further validated the regulatory role of CeO_2_. CeO_2_–IrO_2_ exhibited distinct Ir^6+^ spectral characteristics, indicating that CeO_2_ was conducive to the oxidation of water to produce protons (Supplementary Figs. 30 and 31). Temporal decoupling between H^+^ formation and Ir oxidation became evident after 1.7 V versus RHE, highlighting a voltage-driven mechanism in which CeO_2_ lowers the activation barrier for proton transfer. This enabled rapid proton conduction along the hydroxyl rich CeO_2_ surface, facilitating efficient charge separation during water oxidation.

Therefore, we propose a spatiotemporally decoupled anti-corrosion mechanism model as illustrated in Fig. 5c. During the initial adsorption stage (<50 fs), the CeO_2_ component preferentially adsorbs water molecules owing to its strong oxophilicity. This facilitates ultrafast electron transfer mediated by oxygen vacancies, driving the oxidation of Ir centres to the +6 oxidation state. In the subsequent charge transfer stage (50–150 fs), water dissociation was triggered to generate *OH intermediates and protons. Concurrently, electron transfer between the catalyst and intermediate led to the reduction of Ir^6+^ to Ir^4+^, as evidenced by the rapid decay of the Ir^6+^ signal within 100 fs. In the subsequent reaction stage (100–300 fs), the absence of coexisting Ir^6+^ species prevented the formation of cationic vacancies, although protons generated via water oxidation persist on the catalyst surface. This temporal mismatch between proton and electron dynamics ensured a consistently high formation energy for Ir vacancies, thereby suppressing Ir dissolution. In the final desorption stage (>300 fs), protons rapidly depart from the surface without accumulating, and the high vacancy formation energy was maintained. This spatiotemporal decoupling effect minimized the overlap of proton and electron populations in both time and space, effectively mitigating anodic dissolution. In contrast, commercial IrO_2_ exhibited residual protons in the early reaction stage (<150 fs) that were not efficiently removed during the previous oxidative cycle. The temporal overlap of these protons with electrons reduced the cationic vacancy formation energy, thereby accelerating Ir dissolution.

## Performance testing of PEMWE under fluctuating power

**Fig. 6:**
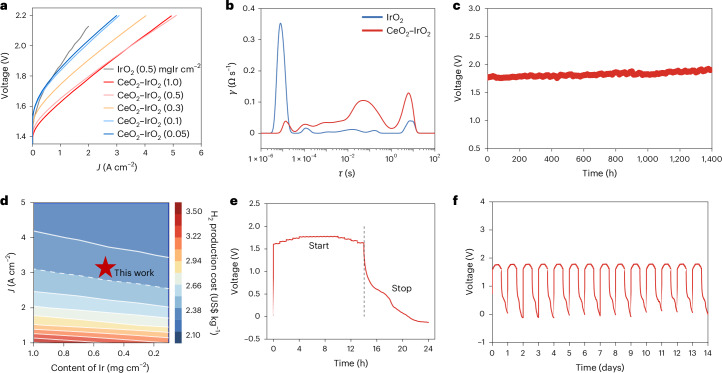
Performance under fluctuating conditions. **a**, The LSVs of CeO_2_–IrO_2_ and IrO_2_ in PEMWE. **b**, The electrochemical impedance spectroscopy distribution of relaxation times of CeO_2_–IrO_2_ and IrO_2_. **c**, A chronopotentiometric stability test of CeO_2_–IrO_2_ at a current density of 1 A cm^−2^. **d**, A techno-economic analysis of CeO_2_–IrO_2_ in proton exchange membrane electrolyser applications. **e**, An evaluation of MEA performance under simulated PV fluctuation conditions. **f**, The proton exchange membrane electrolysis stability under fluctuating power input simulating real-world PV operation. Source data

The microscopic temporal decoupling of proton and electron dynamics effectively suppresses both the reduction in cationic vacancy formation energy and Ir dissolution, leading to enhanced stability of CeO_2_–IrO_2_ in PEMWE. The CeO_2_–IrO_2_ membrane electrode delivered outstanding performance even at reduced iridium loading (0.5 mg cm^−2^) (Fig. 6a). At a high current density of 3 A cm^−2^, the cell required only 1.93 V, substantially lower than the voltage needed by commercial IrO_2_ under the same conditions. The electrochemical impedance spectroscopy distribution of relaxation times analysis revealed a pronounced decrease in impedance at high frequencies, suggesting a substantial enhancement in proton-transfer kinetics (Fig. 6b). The incorporation of CeO_2_ in the membrane electrode under operating conditions effectively suppressed the red shift of OH vibrational modes, indicating enhanced proton transfer and mitigation of local acidification due to proton accumulation (Supplementary Figs. 32 and 33). Long-term durability testing demonstrated the catalyst’s exceptional operational stability, maintaining continuous electrolysis at 1 A cm^−2^ for about 1,400 h without performance degradation (Fig. 6c).

Techno-economic analysis indicated that after coupling with low-cost renewable electricity, CeO_2_–IrO_2_ offered the advantages of high current density and low-Ir loading, enabling a hydrogen production cost less than US$2.4 kg^−1^ (Fig. 6d and Supplementary Figs. 34 and 35). With its rapid proton transport kinetics, the system effectively maintains dynamic stability under the fluctuating power conditions typical of renewable energy sources. Electrochemical start–stop tests showed that the CeO_2_-enhanced catalyst maintained a rapid voltage response on the timescale of seconds (Supplementary Fig. 36). In accelerated ageing experiments, the catalyst remained operational for 37,385 start–stop cycles at 500 mA cm^−2^. Simulated photovoltaic (PV)-driven operating conditions further validated the catalyst’s stability (Fig. 6e). Under a regime of daily stepped power loading (20% to 100% to 20% state of charge) alternating with nighttime shutdown, the CeO_2_–IrO_2_ system maintained stable performance over 14 days of continuous cycling, confirming its practical suitability for renewable-integrated hydrogen production (Fig. 6f).

## Conclusion

We introduced Lewis acidic CeO_2_ onto IrO_2_ to regulate the sequence and kinetics of proton–electron transfer on the femtosecond timescale, enabling stable catalysis with reduced Ir loading. Experimental results revealed that CeO_2_ accelerated proton transfer and decoupled proton and electron transfer into a temporally asynchronous pathway (electrons <100 fs and protons >100 fs) to effectively suppress Ir oxidation and corrosion. The CeO_2_–IrO_2_ catalyst achieved an industrially relevant performance of 3 A cm^−2^ at 1.93 V with a low Ir loading of 0.5 mg cm^−2^, maintaining stability for about 1,400 h in PEMWE. This work offers a time-resolved design paradigm for developing low Ir PEMWE catalysts that meet industrial demands. Furthermore, a femtosecond timescale mechanistic perspective is provided for investigating complex multiphase electrochemical systems, including CO_2_ reduction and liquid phase batteries.

## Methods

### fs-ECTAS measurements

fs-ECTAS measurements were conducted using a custom designed in situ electrochemical optical platform. The working electrode was prepared by uniformly spray coating the target sample onto a fluorine-doped tin oxide conductive glass substrate. The electrolyte was continuously circulated through the electrochemical cell using a peristaltic pump to ensure stable operation. Counter and reference electrodes were symmetrically positioned on the non-optical regions flanking the working electrode, thereby minimizing interference with optical measurements. A femtosecond laser source was split into pump and probe beams using a beam splitter. The pump beam was directed through a tunable optical delay line and focused onto the surface of the working electrode to excite the sample. Simultaneously, the probe beam monitored transient changes in optical absorption in real time. A high-precision potentiostat applied a constant bias voltage to the system, and the transient absorption signals were recorded as a function of pump–probe delay. This setup enabled the extraction of excited-state kinetic information under steady-state electrochemical conditions.

### In situ UV–vis measurements

The in situ UV–vis absorption spectroscopy was conducted using a custom transmission type absorption cell, which was compatible with the Shimadzu UV-2600 UV–vis absorption spectrometer in use. The sample was sprayed onto the fluorine-doped tin oxide as the working electrode and placed on the optical path. The working electrode was made of Pt wire, and the reference electrode was Ag/AgCl. During the scanning test of the linear scanning curve (LSV), the ultraviolet spectra were simultaneously collected in real time. The time-resolved test adopted the kinetic test method, and the real-time measurement was carried out at the position of maximum absorption. The electrochemical signal was collected by multiple acquisitions of 10 s pulse and 10 s stop.

### PEMWE measurement

MEAs for PEMWE were prepared using Nafion 115 membranes and the catalyst prepared in our laboratory. Catalyst-coated membranes were fabricated by sequential spray coating five uniform layers with each containing 0.1 mgIr cm^−2^ catalyst loading. The mass of each sprayed layer was adjusted according to the Ir proportion. It resulted in a total Ir loading of 0.5 mg cm^−2^ on the anode. The cathode employed a commercial Pt/C catalyst (0.1 mg cm^−2^). Bare carbon paper and Pt-coated titanium mesh were used as the gas diffusion layers on the cathode and anode sides, respectively. The assembled MEAs were tested in a custom proton exchange membrane electrolyser using a continuous flow of deionized water without added electrolyte.

## Online content

Any methods, additional references, Nature Portfolio reporting summaries, source data, extended data, supplementary information, acknowledgements, peer review information; details of author contributions and competing interests; and statements of data and code availability are available at 10.1038/s41565-026-02136-x.

## Supplementary information

Supplementary Information Supplementary Methods, Notes 1–4, Figs. 1–36 and references.

## Source data

Source Data Fig. 1 Statistical source data.

Source Data Fig. 2 Statistical source data.

Source Data Fig. 3 Statistical source data.

Source Data Fig. 4 Statistical source data.

Source Data Fig. 5 Statistical source data.

Source Data Fig. 6 Statistical source data.

## Data Availability

The data supporting the findings of this study are available within the paper and Supplementary Information. Source data are provided with this paper.
