# Supplementary material for: Proton–electron temporal asynchrony on femtosecond timescales enables anti-corrosive low-iridium anodes for PEM electrolysers
Source: Nat Nanotechnol. 2026 Feb 27;21(4):598–605. doi: 10.1038/s41565-026-02136-x (PMC13106038; doi:10.1038/s41565-026-02136-x)
Supplement: Supplementary file 1 — Supplementary Methods, Notes 1–4, Figs. 1–36 and references. [file 41565_2026_2136_MOESM1_ESM.pdf]

nature nanotechnology

Supplementary information https://doi.org/10.1038/s41565-026-02136-x

Proton–electron temporal asynchrony on femtosecond timescales enables anti-corrosive low-iridium anodes for PEM electrolysers

In the format provided by the authors and unedited

Table of contents

Supplementary Methods 3
Materials 3
Characterizations 3
Electrochemical measurements 3
Rotating ring-disk electrode (RRDE) measurements 4
Computational details 5
In situ differential electrochemical mass spectrometry (DEMS) measurements 5
In situ Raman measurements 6
In situ XRD measurements 6
In situ electrochemical quartz crystal microbalance (EQCM) measurements 6
Techno-economic analysis (TEA) details 6
Supplementary Note 9
Supplementary note 1. Selection of CeO_2-IrO_2 9
Supplementary note 2. Structural Characterization of CeO_2-IrO_2 9
Supplementary note 3. The Performance of CeO_2-IrO_2 10
Supplementary note 4. In situ Raman spectroscopy of membrane electrodes 10
Supplementary Figures 12
Supplementary Figure 1. 12
Supplementary Figure 2. 12
Supplementary Figure 3. 13
Supplementary Figure 4. 13
Supplementary Figure 5. 14
Supplementary Figure 6. 14
Supplementary Figure 7. 15
Supplementary Figure 8. 15
Supplementary Figure 9. 16
Supplementary Figure 10. 16
Supplementary Figure 11. 17
Supplementary Figure 12. 17
Supplementary Figure 13. 18

Supplementary Figure 14. 18
Supplementary Figure 15. 19
Supplementary Figure 16. 19
Supplementary Figure 17. 20
Supplementary Figure 18. 20
Supplementary Figure 19. 21
Supplementary Figure 20. 21
Supplementary Figure 21. 22
Supplementary Figure 22. 22
Supplementary Figure 23. 23
Supplementary Figure 24. 23
Supplementary Figure 25. 24
Supplementary Figure 26. 24
Supplementary Figure 27. 25
Supplementary Figure 28. 25
Supplementary Figure 29. 26
Supplementary Figure 30. 26
Supplementary Figure 31. 27
Supplementary Figure 32. 27
Supplementary Figure 33. 28
Supplementary Figure 34. 28
Supplementary Figure 35. 29
Supplementary Figure 36. 29
Supplementary References 30

Supplementary Methods

Materials

Iridium(III) chloride hydrate ($IrCl_3 \cdot 3H_2O$, 0.18 mmol) and cerium(III) nitrate hexahydrate ($Ce(NO_3)_3 \cdot 6H_2O$, 0.02 mmol) were dissolved in 5 mL of deionized water under magnetic stirring for 30 min to form a homogeneous solution. A separately prepared aqueous solution containing sodium hydroxide (NaOH, 5 mmol) and sodium nitrate ($NaNO_3$, 5 mmol) in 5 mL of water was then added dropwise to the above mixture, followed by continuous stirring for additional 2 h. The resulting mixture was transferred to a drying oven and kept at 100 ℃ for 24 h to evaporate the solvent. The dried solid was then subjected to calcination in a muffle furnace at 800 ℃ for 8 h under static air, with a heating rate of 10 ℃ min^{-1}. The obtained black powder was washed three times with deionized water by ultrasonic dispersion and centrifugation, and finally dried at 100 ℃ for 12 h. The control sample, IrO_2, was synthesized using the same procedure but without the addition of cerium (III) nitrate. The synthesis of MO_x-IrO_2 followed the same procedure as that of CeO_2-IrO_2, using the corresponding metal salts (e.g., $CrCl_3 \cdot 6H_2O$, $Na_2MoO_4 \cdot 2H_2O$, $Na_2WO_4 \cdot 2H_2O$, $NaVO_3 \cdot 2H_2O$, $NbCl_5$, $TaCl_5$). The molar ratio of M to Ir was kept consistent with that used in the synthesis of CeO_2-IrO_2.

Characterizations

X-ray diffraction (XRD) measurements were carried out on a Rigaku D/Max-2400 diffractometer with Cu Kα radiation (λ = 0.1542 nm) under a constant voltage of 40 kV. Transmission electron microscopy TEM/high resolution (HR)-TEM images and energy dispersive X-ray spectroscopy (EDX) mapping of samples were obtained on a Tecnai G2 F30 filed emission TEM. Atomic-scale high-angle annular dark field scanning transmission electron microscopy (HAADF-STEM) images were recorded on a probe aberration-corrected STEM (Cubed Titan G2 60-300, FEI, USA) operated at 300 kV. X-ray absorption spectroscopy (XAS) spectra were collected on the Rapid XAFS 2M (Anhui Absorption Spectroscopy Analysis Instrument Co., Ltd.) in transmission (or fluorescence) mode at 20 kV and 20 mA. X-ray photoelectron spectroscopy (XPS) analysis was performed on a Kratos Axis Ultra with a Delay Line Detector photoelectron spectrometer using an Al monochromatic X-ray source. All XPS spectra were corrected using C 1*s* line at 284.8 eV.

Electrochemical measurements

Electrochemical experiments were performed in 0.5 M H_2SO_4 on a CHI-760E Electrochemical Workstation (CHI Instruments) using a standard three-electrode system. Before the electrochemical measurement, the electrolyte was degassed by bubbling oxygen for at least 30 min to achieve a saturation

condition of oxygen. All electrochemical investigations were performed at 25 ℃ using a Hg/Hg_2SO_4 as a reference electrode, a Pt plate as the counter electrode, and carbon fibers with an area of 0.5 × 2 cm loaded with a catalyst as the working electrode in 0.5 M H_2SO_4, unless otherwise specified. The 8 mg catalyst and the 20 μL Nafion solution (5 wt%) were dispersed in 2 mL isopropanol and water (v/v, 1:1) mixed solution. It sonicated in ice water for 30 minutes to get a homogeneous ink. The ink was subjected to high power cell pulverization ultrasonication for 4 hours. The catalyst ink was sprayed onto the carbon fibers to achieve the desired loading. LSV plots measurements were set at 10 mV s^{-1} for peroxidation test. Also, the EIS tests were performed at open-circuit potentials (OCP) in the frequency range of 0.01-100 kHz. The potentials were corrected to compensate for the effect of solution resistance and calculated using the following equation:

E_{iR} corrected = E - iR

where R is the uncompensated ohmic solution resistance measured via iR compensation module in CHI760E with comp level of 95 %.

Rotating ring-disk electrode (RRDE) measurements

The pH value on the surface of disk electrode can be determined by monitoring the OCP changes on IrO_x-modified ring electrode[1-4]. IrO_x was firstly electro-deposited onto Pt-ring electrode of RRDE by CV cycling (-0.45-0.75 V vs. Ag/AgCl, 1.0 V s^{-1}, 300 cycles starting in the negative direction). Electrolyte for the electrodeposition was prepared as reported and was saturated using Ar gas before and during electrodeposition[5-7]. Then, the OCP (E_{OC}) of the IrO_x-modified ring electrode was recorded in 0.5 M K_2SO_4 electrolyte at different pH, which was adjusted by gradually adding H_2SO_4. The pH dependence relationship of the E_{OC} was established by linear fitting. The pH_{ring} values were obtained by converting E_{OC} using the following equation:

$$pH_{ring} = -(E_{OC} - b)/a \quad (1)$$

where a and b are the slope and intercept values of the fitted line, respectively. The pH evolution upon catalyst surface during OER was measured in Ar-saturated alkaline saline. 10 μL of the abovementioned CeO_2-IrO_2 ink was dropped onto the disk electrode. LSV was performed on the disk electrode at a scanning rate of 1 mV s^{-1}, and E_{OC} of the IrO_x-modified ring electrode was simultaneously recorded[8-10]. The pH_{ring} of the IrO_x-modified ring was calculated from the E_{OC} using equation (1). Then the pH_{disk} of the catalyst-loaded disk electrode was calculated based on the pH_{ring} using the following equation:

$$C_{H+,ring} - C_{OH-,ring} = N_D (C_{H+,disk} - C_{OH-,disk}) + (1 - N_D)(C_{H+,bulk} - C_{OH-,bulk}) \quad (2)$$

where C_m is the concentration of species *m* on the ring or disk electrode, or in the bulk electrolyte, respectively; N_D is defined as the detection efficiency and was calculated as 0.36.

Computational details

Excited-state energy was performed using the CP2K 2025.1 software package. The IrO_2 (110) surface was modeled as a periodically repeated slab with a vacuum layer of at least 15 Å. Structures of H-adsorbed and OH-adsorbed IrO_2 (110) were fully optimized using spin-polarized (unrestricted Kohn-Sham, UKS) DFT with the PBE functional and D3 dispersion correction with Becke-Johnson damping [D3(BJ)] [11]. The bottom two atomic layers were fixed to bulk-like positions, while the top layers and adsorbates were allowed to relax. Core electrons were described by Goedecker-Teter-Hutter (GTH) pseudopotentials, and valence electrons were expanded in the DZVP-MOLOPT-SR-GTH basis set [12]. The Gaussian and Plane Waves (GPW) method was employed with a plane-wave density cutoff of 400 Ry, a relative cutoff of 60 Ry, and an SCF convergence tolerance of 1.0×10^{-6} a.u. Geometry optimizations were converged until the maximum atomic force was below 4.5×10^{-4} a.u. Excited-state properties were computed via linear-response TDDFT using the PBE0 hybrid functional (25% exact exchange), also within a UKS framework to account for open-shell character where present. The lowest 50 singlet excited states were calculated to ensure well-converged spectral profiles in the UV-visible range. Transition energies and oscillator strengths were extracted and processed with Multiwfn 3.8 dev to generate simulated absorption spectra (Gaussian broadening, full width at half maximum = 0.2 eV) [13]. The hole formation energy was carried out with the Vienna Ab Initio Simulation Package (VASP) code[14]. The ion-electron interactions were represented by the projector-augmented wave (PAW) method and the electron exchange and correlation were treated with the generalized gradient approximation (GGA) within Perdew, Burke, and Ernzerhof (PBE) functional[15,16]. The cut-off energy for plane wave is set to 400 eV. All the structures are relaxed until the residual forces on the atoms have declined to less than 0.05 eV $Å^{-1}$. The IrO_2 (110) surface models consist of periodic 3×3 four-layer slabs with a vacuum separation of 15 Å in the direction perpendicular to the surface. The two bottom layers were fixed while the remaining top layers and the adsorbates were allowed to fully relax.

In situ differential electrochemical mass spectrometry (DEMS) measurements

The reaction products of OER process were measured using DEMS (QAS100 device, Shanghai Linglu Instrument). The saturated Ag/AgCl electrode and Pt line are used as reference electrode and counter electrode, respectively[17-22]. The working electrode is prepared by sputtering Au on a 50-μm-thick, porous polytetrafluoroethylene film. Then, the catalyst is dropwise injected onto Au with a loading mass of 1 mg cm^{-2}. LSV measurements were conducted in 0.5 M H_2SO_4 solution at a scan rate of 5 mV s^{-1}, with the potential versus RHE ranging from 1.2 V to 1.8 V. The real-time changes in oxygen isotope masses 32, 34, and 36 were recorded during the test.

In situ Raman measurements

The in-situ Raman spectroscopy measurement of membrane electrode working conditions was carried out on the LabRAM HR Evolution spectrometer with the excitation wavelength of 532 nm. Using a visualized membrane electrode electrolytic cell, the Raman light was focused onto the catalyst surface through the visualization window to obtain the surface chemical composition and structural information of the material.

In situ XRD measurements

In situ XRD is measured by by X-ray diffractometer (XRD, Rigaku Ultima IV) using Cu Kα radiation (λ = 1.54059 Å, 50.0 mA, 40 kV) at the scanning speed of 2 °/min from 30- 70 °. The XRD cell uses a customized three electrode cell, with the sample on Carbon paper as the working electrode, Pt as the counter electrode, and Ag/AgCl as the working electrode. The corresponding potential and frequency signals are collected at 0.5 M H_2SO_4. In situ electrochemical characterization was carried out at specific potential for 20 minutes to obtain the crystal structure of the material information of materials.

In situ electrochemical quartz crystal microbalance (EQCM) measurements

For EQCM measurements, the system was assembled in a temperature-controlled eCell microchamber (Hettich, Germany) connected to an EQCM 15M (Gamry, USA). An AT-cut quartz crystal with a fundamental frequency of 9.12 MHz ± 50 kHz was used. The Au polycrystals coated with thin films of catalysts by spin coating deposition were used as a working electrode. The working electrode was then mounted into a three-compartment electrochemical cell. A Hg/HgO electrode and a Pt wire were used as the reference electrode and counter electrode, respectively. The frequency change was measured by a Q-sensor analyser (QE 401) equipped with the QEC 401 electrochemistry module. The frequency transition signal of the potential is obtained through steady-state polarization and cyclic voltammetry. EQCM results were collected using Gamry Resonator software. For the treatment of data, the following Sauerbrey equation was used to determine the deposited mass based on the parallel frequency (fp) [23-29].

$$\Delta f = -C_f \times m$$

Δf: Frequency variation. C_f: Correction factor (theoretical correction factor for 5 MHz chips: 56.6 Hz cm^2)/μG. m: Quality changes occurring on the surface of the chip.

Techno-economic analysis (TEA) details

To evaluate the economic potential of electrocatalytic hydrogen synthesis using sustainable electricity and pure water as raw material, a 1 MW PEMWE was used as the model. Sustainable electricity is used as an energy supply to get hydrogen from pure water splitting. The cost parameters of the electrolytic

cell come from the equipment company. To calculate the cost of hydrogen under optimal conditions, a daily production rate of 388.8 kg day^{-1} was considered [30-34]. The specific assumptions made for this analysis are outlined below (some of the numbers are constant values calculated beforehand):

1. The system scale is 1 MW.
2. The price range of green power is 0.02-0.05 USD kWh^{-1}.
3. Total current = hydrogen weight × 111672.454/energy efficiency.
4. The cost of the PEMWE stack per square meter amounts to approximately 9000 USD, encompassing 53 % for cell manufacturing, 24 % for anode/cathode and diaphragm, 17 % for porous transport layer, 3 % for small parts, 3 % for stack assembly and end plates.
5. Electrolysis electricity = hydrogen weight × electricity price × stack voltage × 24 × 111.672454/energy efficiency.
6. Total stack area = hydrogen weight × 111.672454/(energy efficiency*current density).
7. Total stack price = hydrogen weight × 111.672454 × stack price/(energy efficiency × current density).
8. Balance of plant = 2.125 × hydrogen weight × 111.672454 × stack price/(energy efficiency × current density).
9. The period of electrode/diaphragm exchange is 5 or 10 years.
10. Total capital cost = 3.12 × hydrogen weight × 111.672454 × stack price/(energy efficiency × current density).
11. (Maintenance + Labor) cost = 3.125 × 0.028 × hydrogen weight × 111.672454 × stack price/(energy efficiency × current density).
12. Water cost = hydrogen weight × 45 × water price.
13. The price of pure water is 0.011 USD kg^{-1}.

The total cost of the proposed sustainable hydrogen preparation process consisted of capital cost, operating cost, and material cost.

Capital Cost:

Total stack price:

Total stack price = Total stack area × Stack price

Balance of Plant:

Balance of plant = Total stack price × Balance of plant ratio/(100-Balance of plant ratio)

Operating Cost:

Electrolysis electricity:

Electrolysis electricity = electricity price $\times$ stack voltage $\times$ Total current $\times$ 24/1000

Maintenance:

Maintenance = (Total stack price + Balance of plant) $\times$ 0.01 $\times$ Maintenance rate/365

Labor:

Labor = CAPEX $\times$ Labor rate $\times$ 0.01/365

Electrode/diaphragm exchange:

Electrode/diaphragm exchange = Total stack area $\times$ Price/Lifetime

Material Cost:

Water cost:

Water cost = Water price $\times$ Water consumption $\times$ Hydrogen weight

Total cost:

H_2-levelized cost (USD kg^{-1}) = Electrolysis electricity + Balance of plant + Total maintenance + Labor cost + Electrode/diaphragm exchange + Water cost

Supplementary Note

Supplementary note 1. Selection of CeO_2-IrO_2

In this study, a series of homogeneously synthesized IrO_2-based composite catalysts were systematically evaluated using a standard three-electrode setup in 0.5 M H_2SO_4 electrolyte (**Fig. 2b**). Among all the tested samples, the CeO_2-IrO_2 composite exhibited the most outstanding electrocatalytic performance. To further elucidate the underlying stability mechanism, we employed ^{18}O isotope labeling to quantitatively correlate oxygen dissolution behavior with proton transport properties (**Figs. S10-S11**). Specifically, the catalysts were deposited onto Au-coated waterproof gas diffusion membranes and subjected to cyclic voltametric activation in $H_2{}^{18}O$. After thorough rinsing with deionized water, the activated electrodes were transferred to $H_2{}^{16}O$ electrolyte for evaluation. By in situ monitoring the potential-dependent intensity ratios of ^{16}O-^{18}O to ^{18}O-^{18}O isotopic peaks, we assessed the lattice oxygen solubility of the catalysts at OER-relevant potentials. This proved that CeO_2 offered a path-dependent environment conducive for rapid proton transport, further promoting overall reaction kinetics.

Supplementary note 2. Structural Characterization of CeO_2-IrO_2

We characterized the structure of CeO_2-IrO_2 to explain the structural origin of the enhanced activity. XRD analysis revealed the coexistence of two distinct crystalline phases with diffraction peaks precisely matching those of CeO_2 (JCPDS #78-0694) and IrO_2 (JCPDS #86-0330) (**Fig. S13**). Elemental mapping via TEM confirmed the surface loading of CeO_2 on IrO_2 nanocrystals, and high-resolution TEM images revealed lattice fringes attributable to both phases (**Fig. S14**). Further atomic scale insights were obtained using HAADF-STEM, which clearly showed CeO_2 nanoparticles anchored on the IrO_2 surface (**Fig. S15**). This unique interfacial configuration was further supported by elemental distribution maps, where Ce signals were spatially distinct from Ir signals suggesting well-defined phase separation and localized structural heterogeneity (**Fig. S16**).

Synchrotron based XAS analysis revealed that the near-edge absorption features of Ir in the CeO_2-IrO_2 composite remained unchanged compared to those of pristine IrO_2 (**Fig. S17**). It indicated that incorporation of CeO_2 does not significantly alter the local coordination environment or oxidation state of Ir. XPS further corroborated this observation, showing that the valence state of Ir remains essentially unchanged upon CeO_2 introduction (**Fig. S18**). A slight shift in the oxygen species distribution was observed, which can be attributed to the contribution of the CeO_2 phase.

Supplementary note 3. The Performance of CeO_2-IrO_2

Dynamic polarization curves demonstrate a positive shift in the corrosion potential of the CeO_2-IrO_2 system compared to the control samples. This shift suggests enhanced resistance to anodic dissolution, thereby confirming the composite's superior corrosion stability in oxidative environments (**Fig. S20**). To clarify the influence of Ce content on catalytic performance, we have supplemented a series of activity and stability tests with varying CeO_2 loadings **(Fig. S21)**. At a fixed Ir loading, the CeO_2 to Ir ratio was systematically adjusted to examine the role of Ce. The LSV results revealed that when the Ce content was lower than that of the target sample (10% relative to Ir). The catalytic activity decreased likely due to the weaker interaction between Ce and Ir at low Ce levels. When the CeO_2 content exceeded 10%, the catalytic activity again declined. It can be attributed to the reduced overall electrical conductivity caused by excessive Ce incorporation. These results suggest that an optimal Ce concentration is required to achieve the highest catalytic activity. Stability tests further showed that catalysts with lower Ce contents exhibited inferior durability, indicating that insufficient Ce incorporation failed to provide adequate corrosion protection. When the CeO_2 content was higher than the optimal ratio, the stability initially improved compared with the low Ce sample, confirming the stabilizing effect of Ce. However, excessive Ce content led to increased cell voltage and accelerated oxidation, ultimately compromising long term durability. In summary, only an appropriate amount of Ce can simultaneously optimize both catalytic activity and stability. This balance originates from the proton electron decoupling strategy discussed in the manuscript, which governs the interaction between CeO_2 and Ir active sites during electrocatalysis.

Supplementary note 4. In situ Raman spectroscopy of membrane electrodes

Through the optical transparent window on the anode side, Raman spectroscopy analysis of the membrane electrode assembly (MEA) condition was conducted using a 532 nm laser to monitor the evolution of species during the water oxidation process in the proton exchange membrane water electrolysis (PEMWE) condition (**Fig. S32**). This was achieved by integrating multiple engineering strategies, including a transparent current collector design, channel broadening, and localized micro-pore customization in the gas diffusion layer. Specifically, the CeO_2-IrO_2 membrane electrode was mounted in a custom-built electrochemical cell equipped with an optically transparent window on the anode side, enabling efficient transmission of both the excitation and scattered Raman light through the current collector region. The widened flow channels provided an open optical pathway for the laser beam to pass through the inter channel space, while a micron scale pore fabricated at the center of the gas diffusion layer allowed the laser to be precisely focused on the catalyst electrolyte interface. This hole allowed direct probing of interfacial changes without significantly affecting the overall electrode performance.

The cell enabled real time in situ Raman monitoring of surface reactions on the membrane electrode under high current densities. This hole allowed direct probing of interfacial changes without significantly affecting the overall electrode performance. The cell enabled real-time in situ Raman monitoring of surface reactions on the membrane electrode under high current densities. The position of the water peak observed in the membrane electrode Raman spectra reflects the actual pH variation at the catalyst surface under operating conditions, highlighting the role of CeO_2 in facilitating proton transport. Commercial IrO_2 showed a significant redshift in the *v* (O-H) band at a current density of just 40 mA cm^{-2}, indicative of rapid interfacial acidification and the accumulation of strongly bound water. In contrast, a comparable spectral shift was not observed for the CeO_2-IrO_2 system until the current density reached 100 mA cm^{-2}, indicating that protons were rapidly conducted away by CeO_2 without significant accumulation (**Fig. S33**).

Supplementary Figures

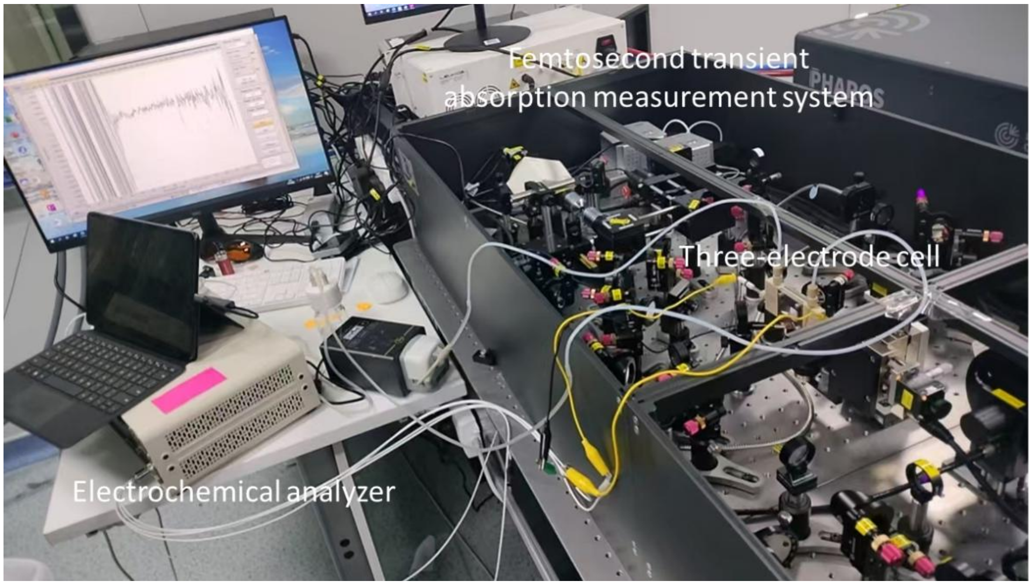

Supplementary Figure 1. The diagram of femtosecond electrochemical transient absorption spectroscopy (fs-ECTAS) device.

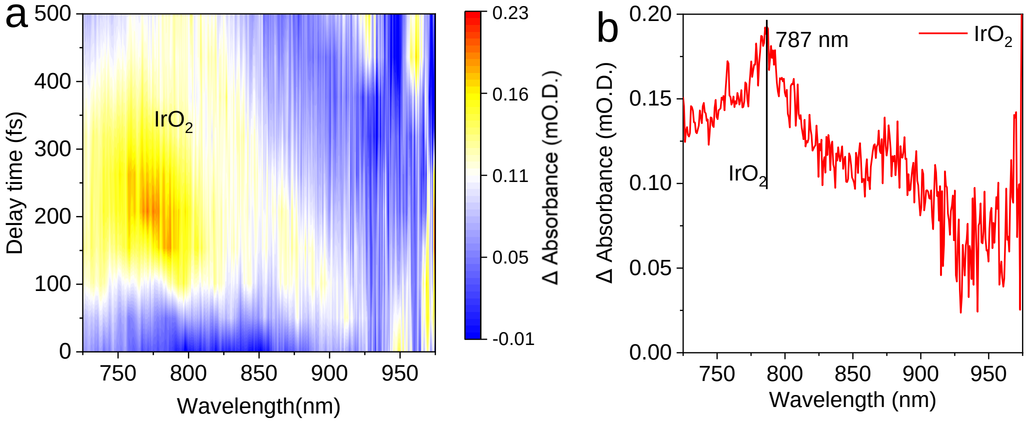

Supplementary Figure 2. a, b, TAS of IrO_2 and specific spectra at 150 fs.

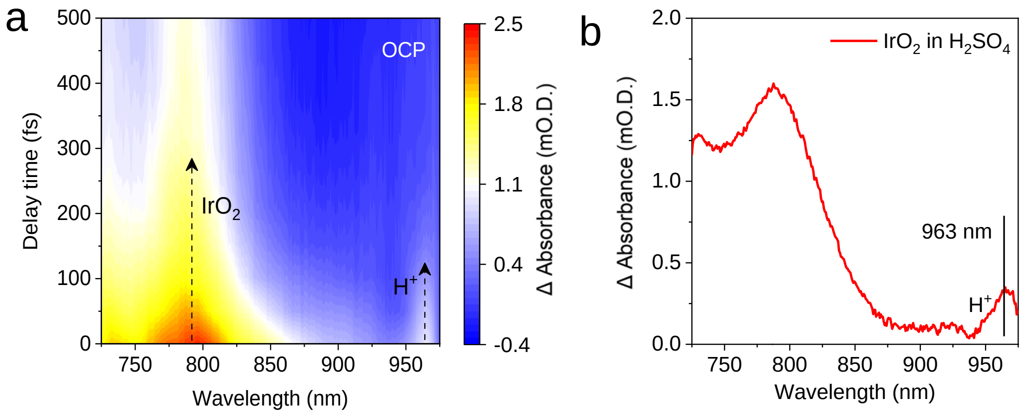

Supplementary Figure 3. a, b, TAS of IrO_2 in H_2SO_4 and specific spectra at 150 fs.

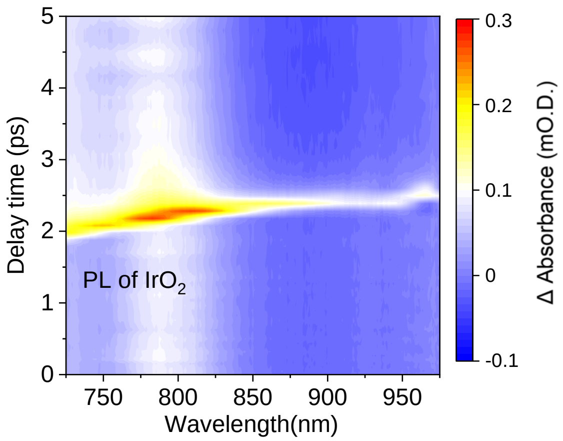

Supplementary Figure 4. The TAS data of IrO_2 before chirp correction.

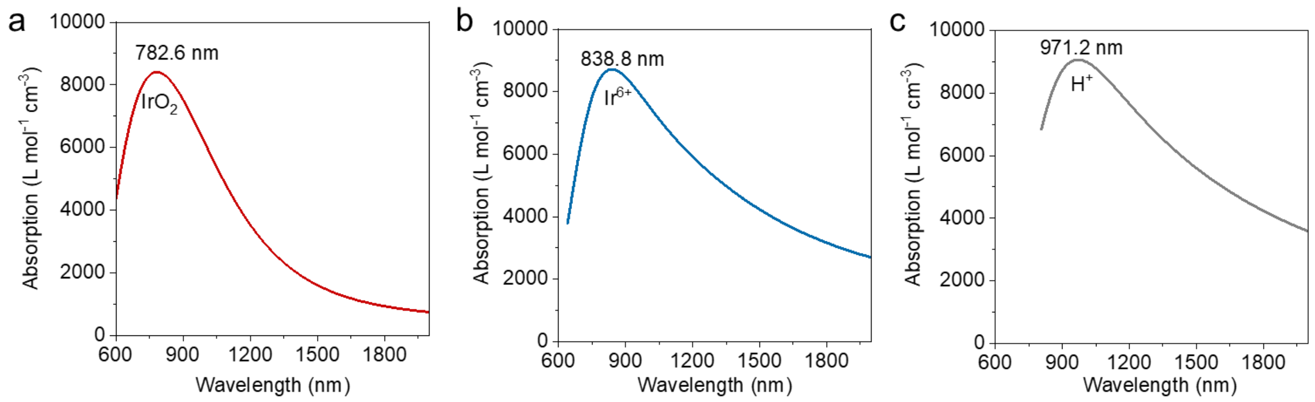

Supplementary Figure 5. a, b, c, DFT calculated absorption spectra of IrO_2, Ir^{6+} and H^+.

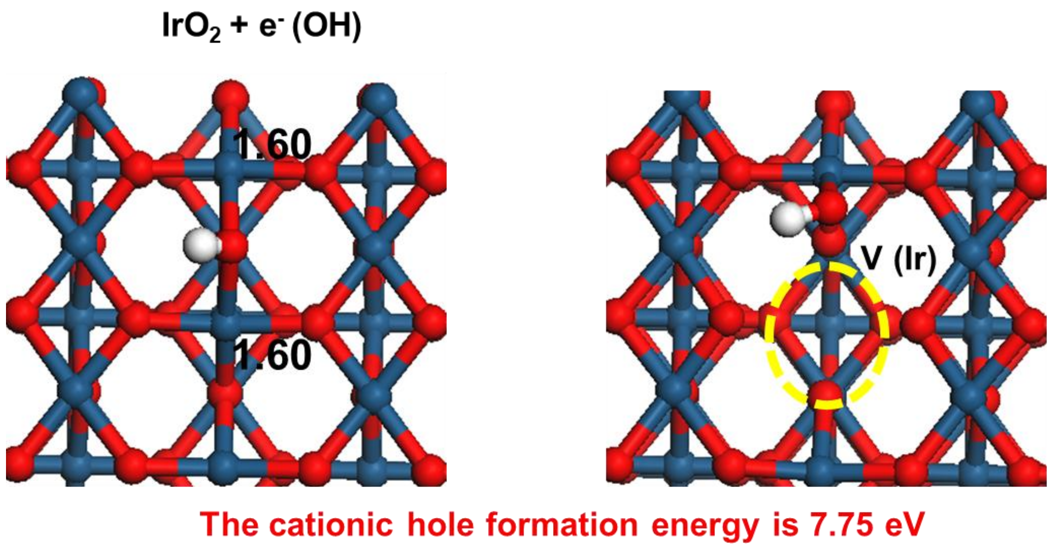

Supplementary Figure 6. The formation energy of Ir cation holes formed by surface OH and electrons.

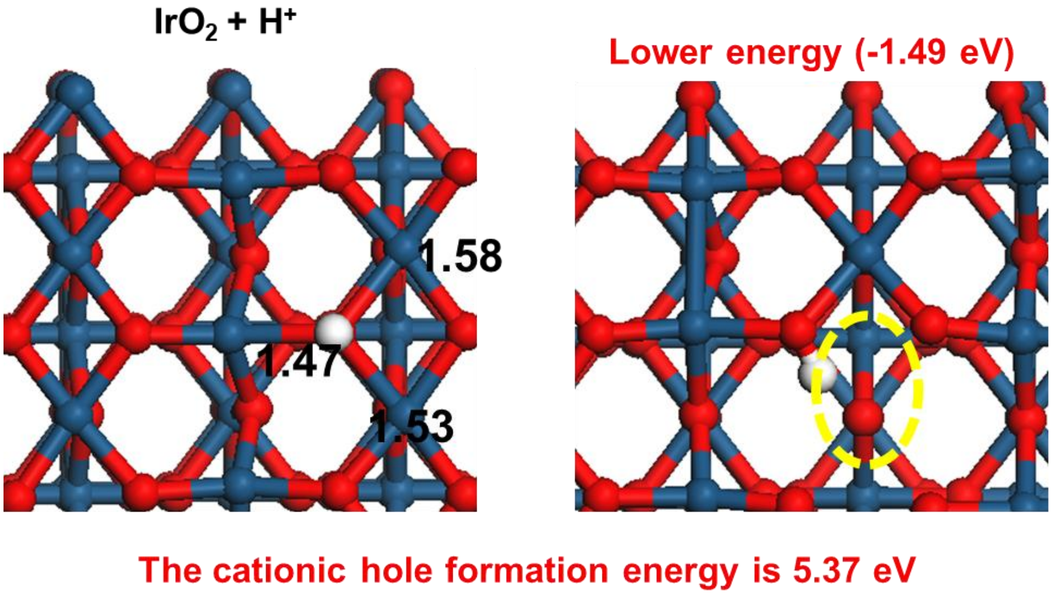

Supplementary Figure 7. The formation energy of Ir cation holes formed by surface H.

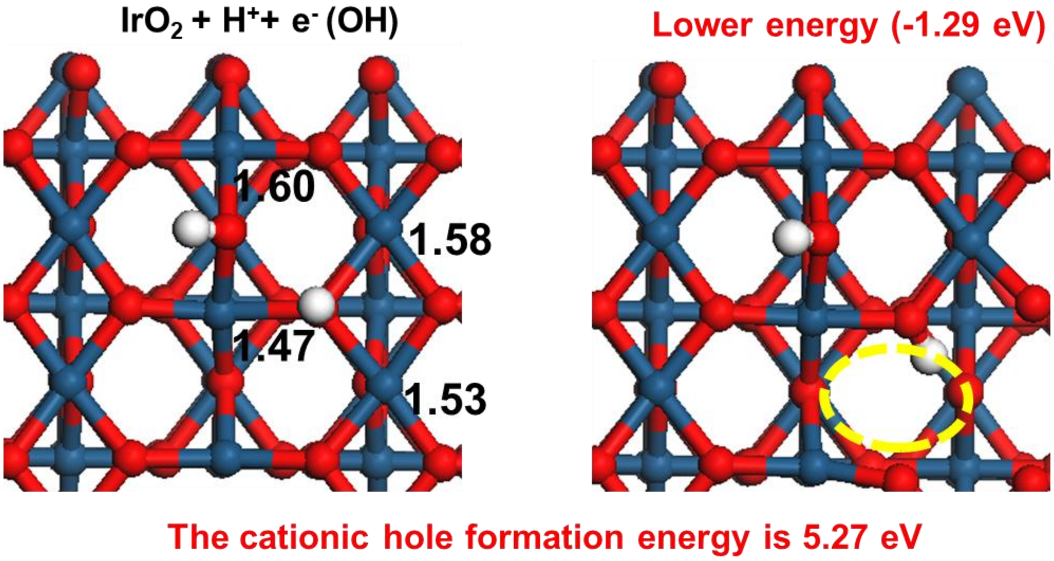

Supplementary Figure 8. The formation energy of Ir cation holes formed by surface H, OH, and electrons.

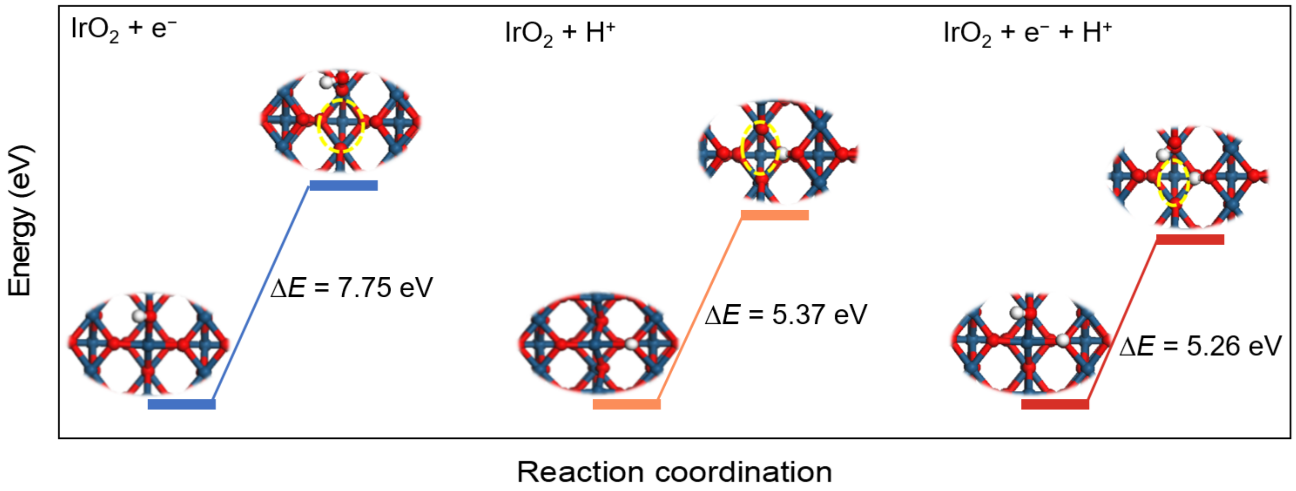

Supplementary Figure 9. Formation energies of Ir cations on commercial IrO_2 surfaces under electron only, proton only, and coupled proton-electron conditions.

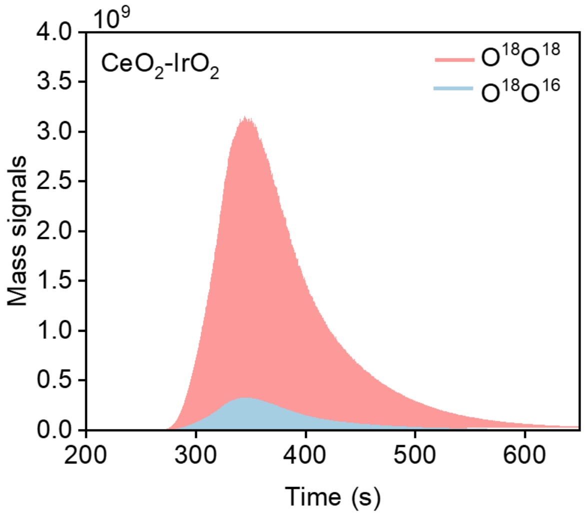

Supplementary Figure 10. Isotopic difference mass spectrometry of CeO_2-IrO_2.

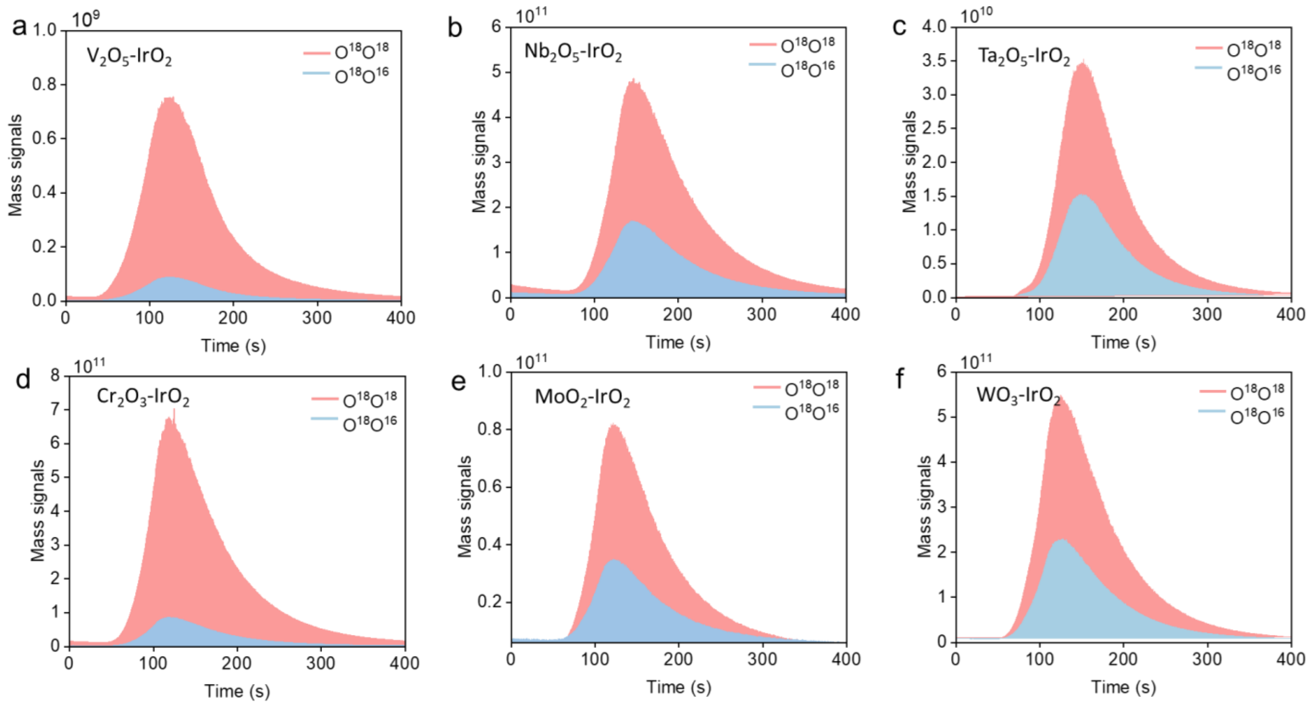

Supplementary Figure 11. a-f, DEMS of (**a**) V, (**b**) Nb, (**c**) Ta, (**d**) Cr, (**e**) Mo and (**f**) W with respect to IrO_2.

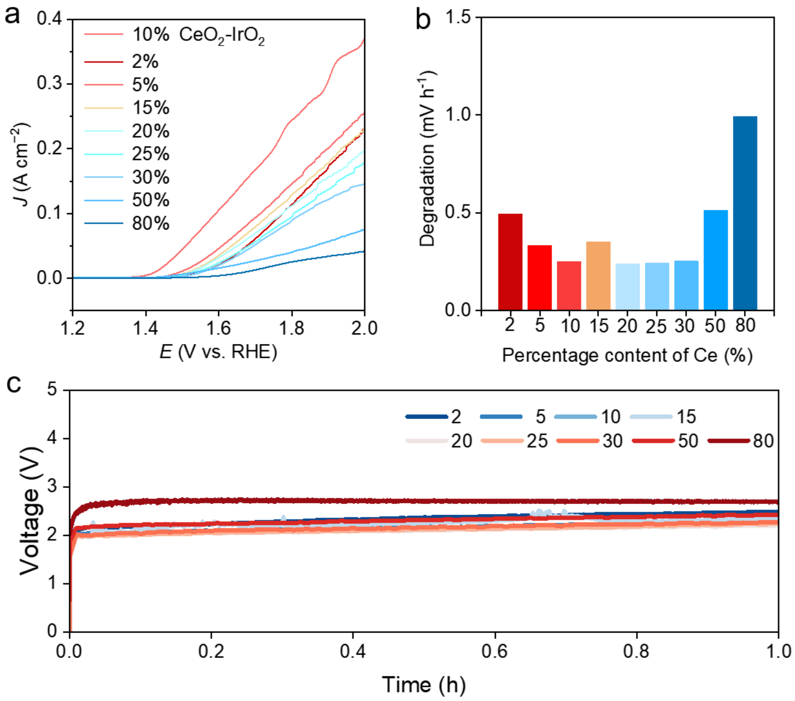

Supplementary Figure 12. a, LSV of CeO_2-IrO_2 with different Ce ratios. **b,** The potential decay rate and **c,** the CP at 200 mA cm^{-2} of CeO_2-IrO_2 with different Ce ratios in H_2SO_4 solution.

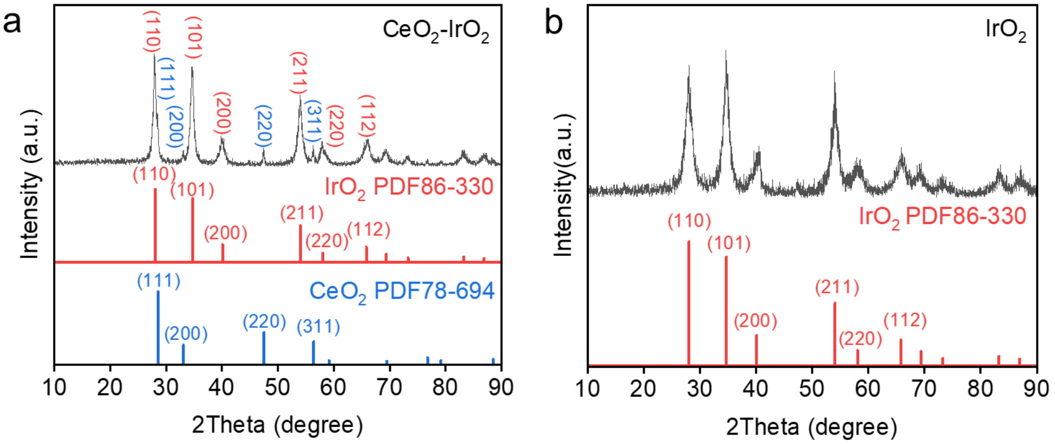

Supplementary Figure 13. a, b, XRD patterns of CeO_2-IrO_2 and IrO_2.

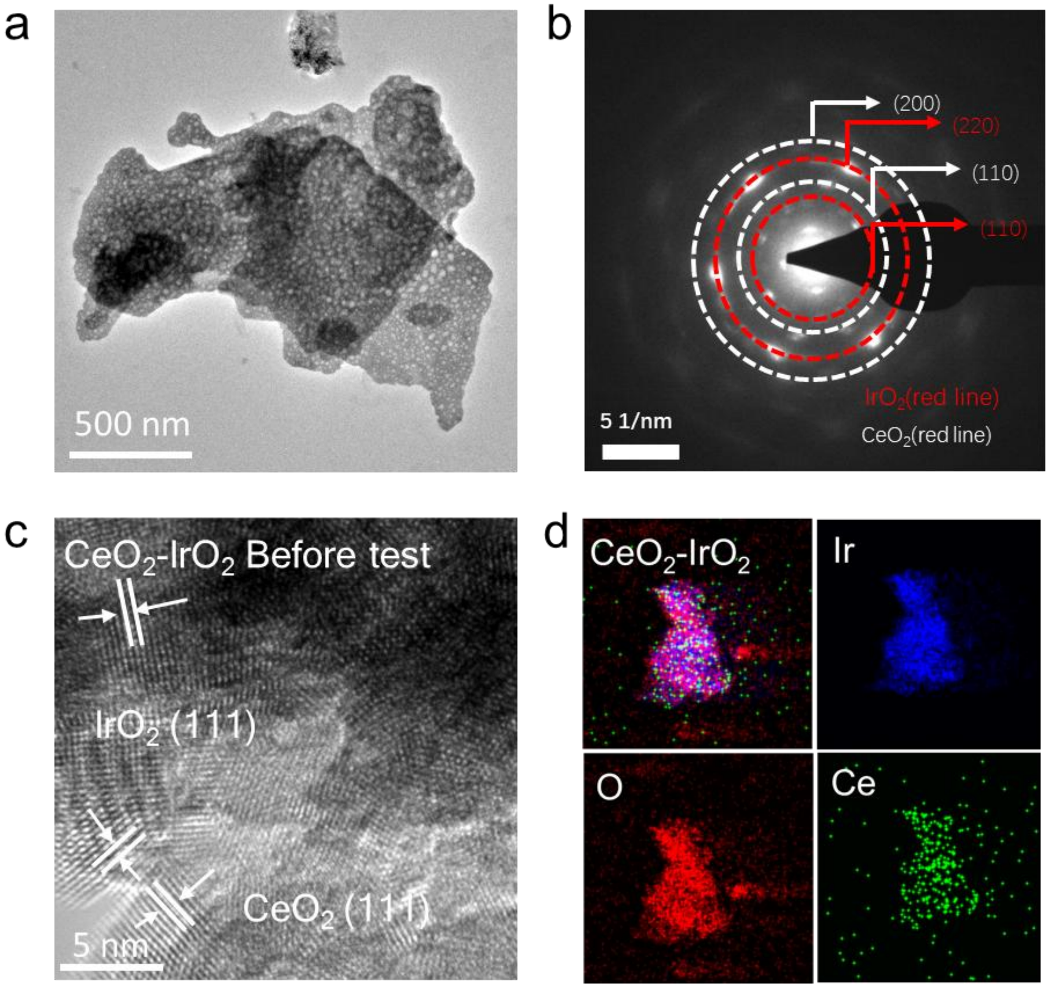

Supplementary Figure 14. a, TEM image, **b,** SAED pattern, **c,** HRTEM, **d,** mapping of CeO_2-IrO_2.

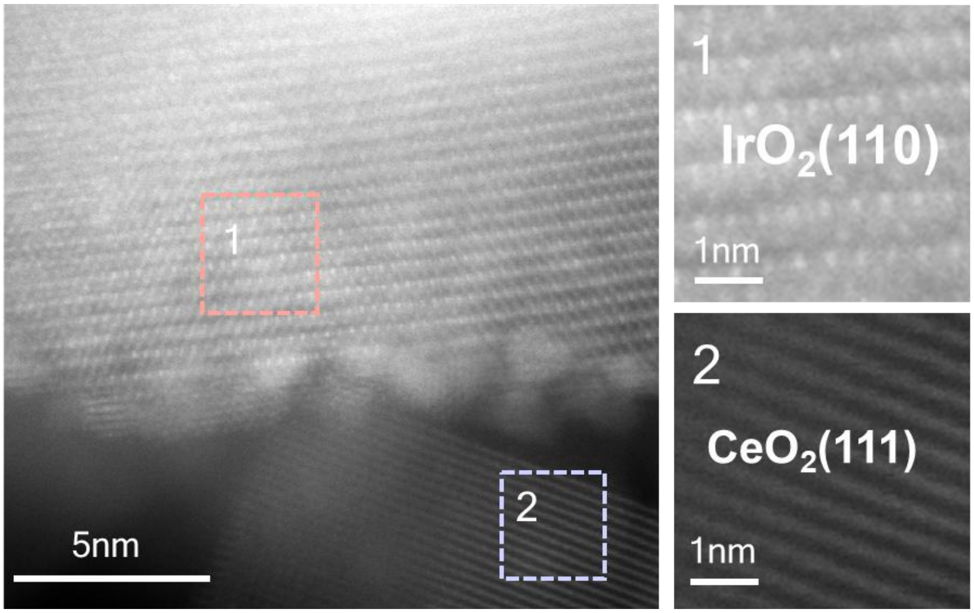

Supplementary Figure 15. The HADDF-STEM image of CeO_2-IrO_2.

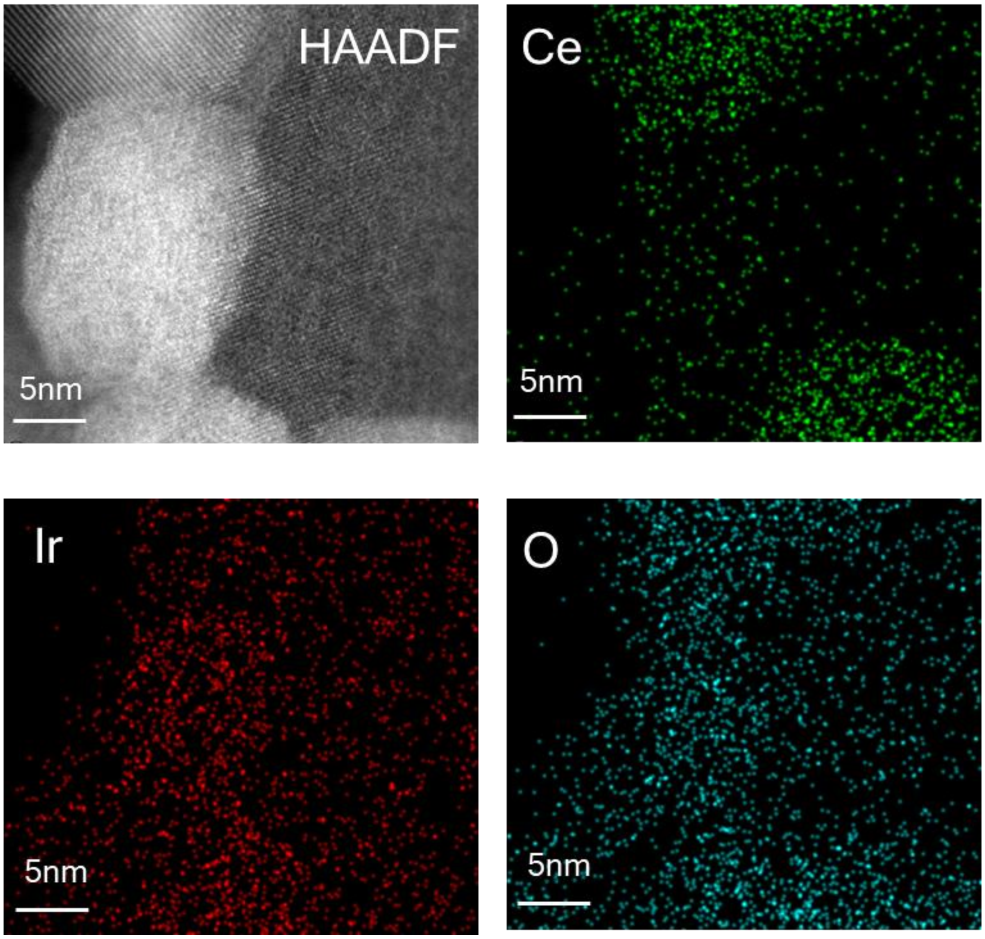

Supplementary Figure 16. The HADDF-STEM elemental mapping image of CeO_2-IrO_2.

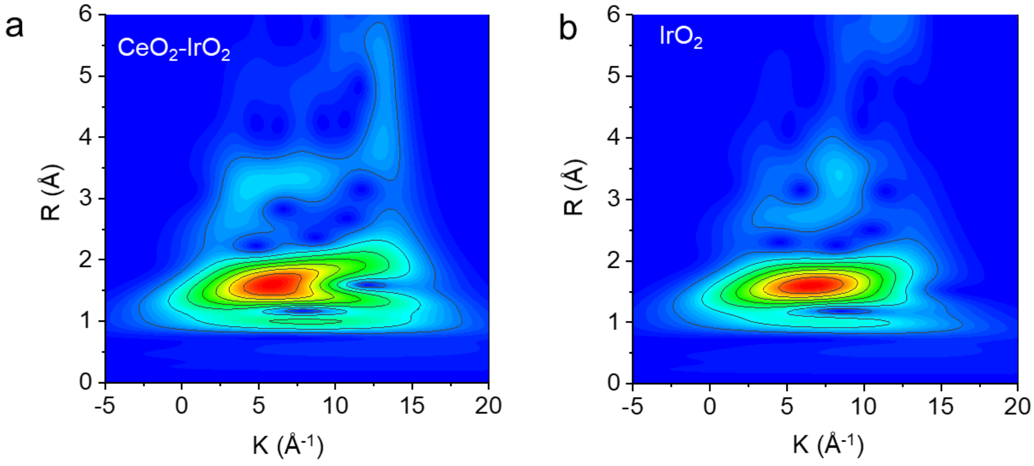

Supplementary Figure 17. a, b, XAS of CeO_2-IrO_2 and IrO_2 in wavelet transform images.

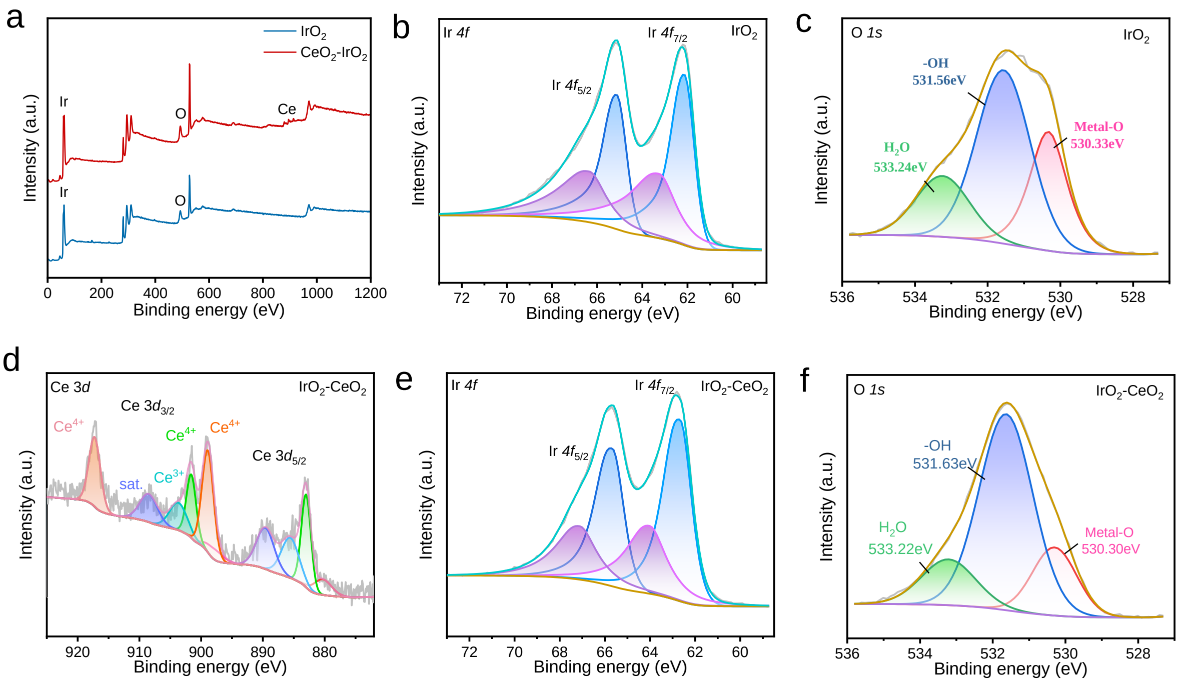

Supplementary Figure 18. a, XPS survey spectra of CeO_2-IrO_2 and IrO_2. **b, c,** O 1*s* and Ir 4*f* of IrO_2. **d, e, f,** Ce 3d, O 1*s* and Ir 4*f* of CeO_2-IrO_2.

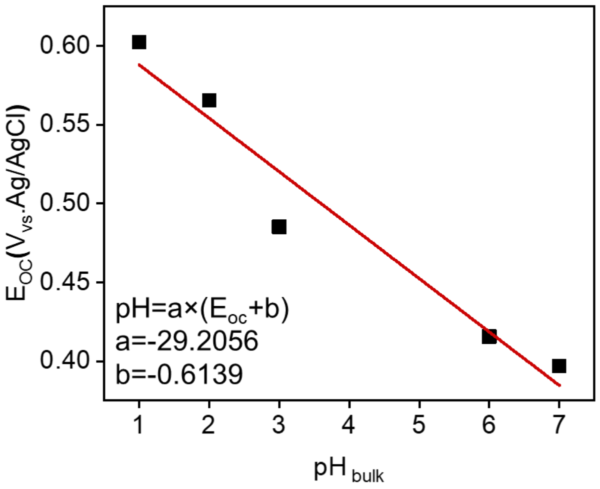

Supplementary Figure 19. RRDE pH calibration standard curve of IrO_x.

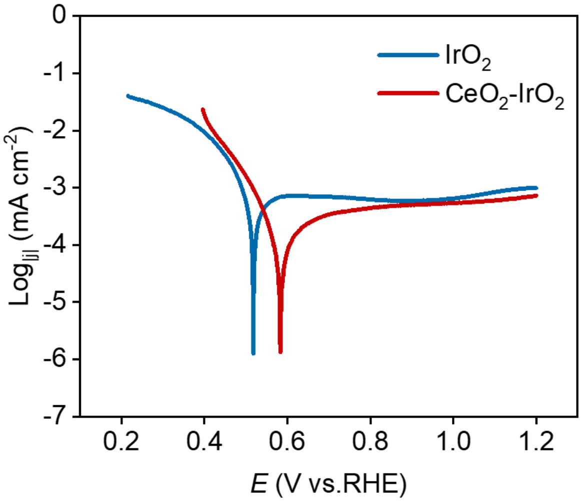

Supplementary Figure 20. Corrosion polarization curves of CeO_2-IrO_2 and IrO_2.

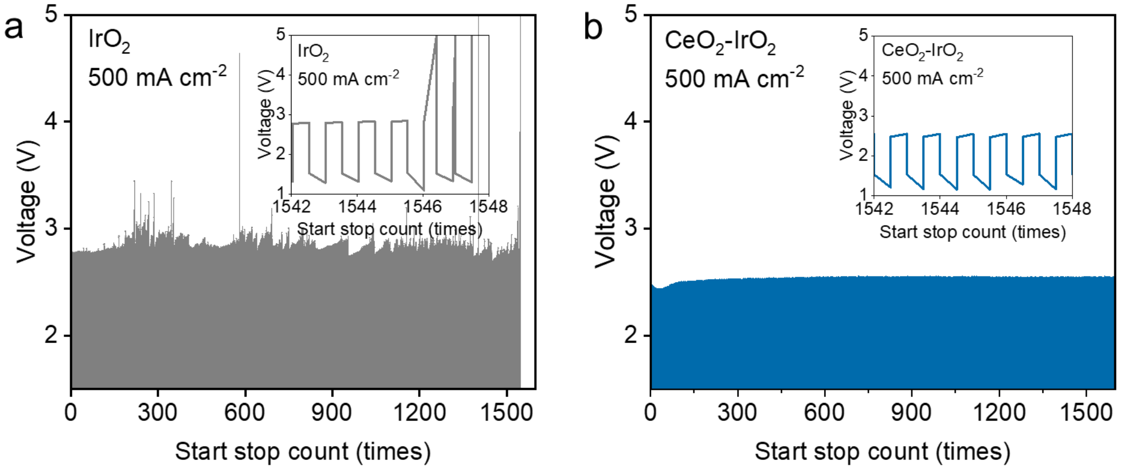

Supplementary Figure 21. Start-stop test of CeO_2-IrO_2 and IrO_2.

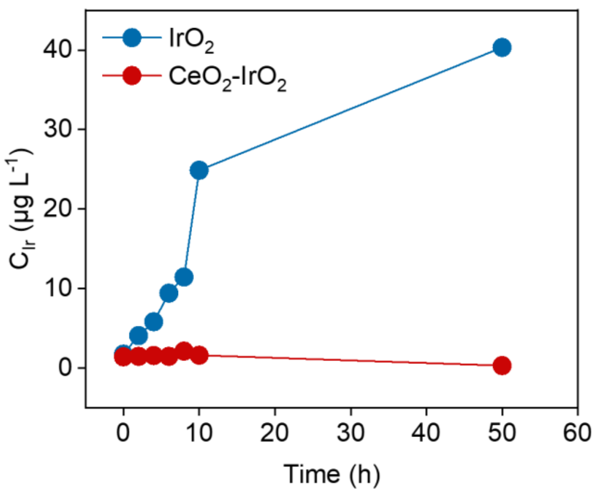

Supplementary Figure 22. ICP analysis of Ir concentration in the electrolyte as a function of time.

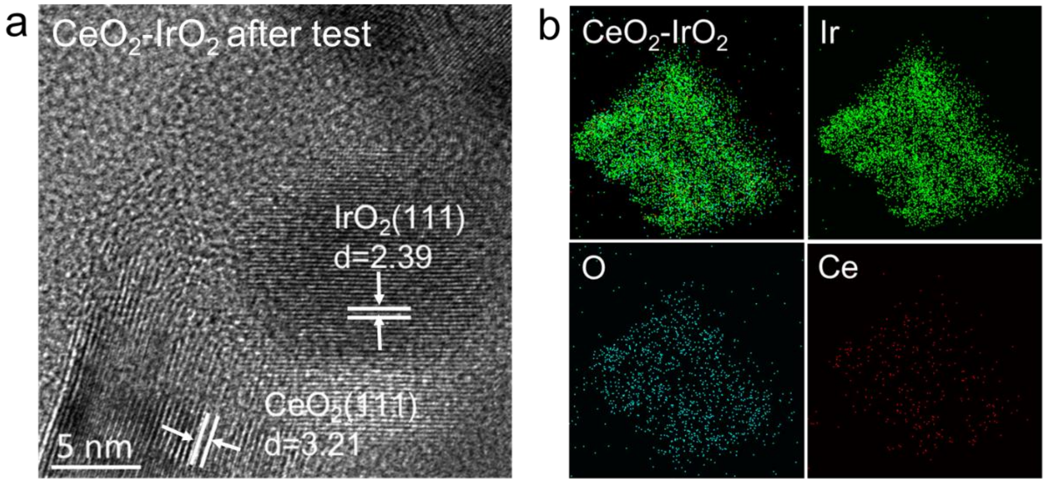

Supplementary Figure 23. a, TEM image, **b,** SAED pattern of CeO_2-IrO_2 after test.

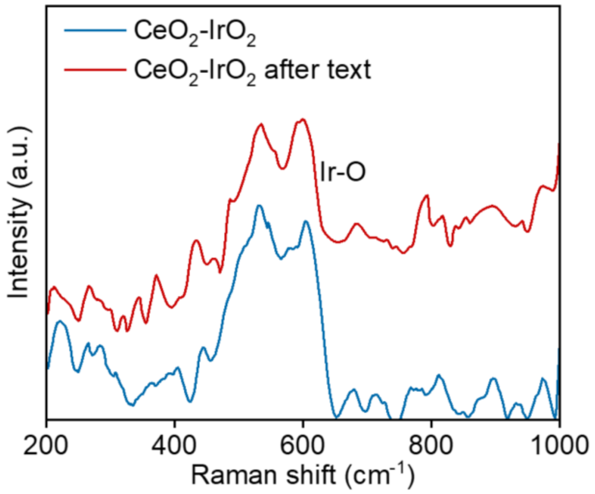

Supplementary Figure 24. Raman of CeO_2-IrO_2 before and after test.

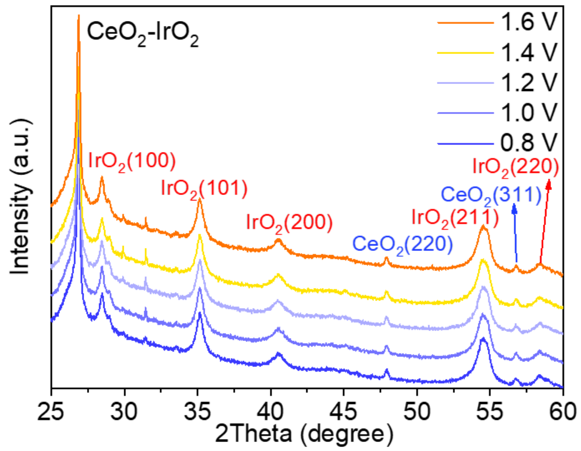

Supplementary Figure 25. In situ XRD of CeO_2-IrO_2.

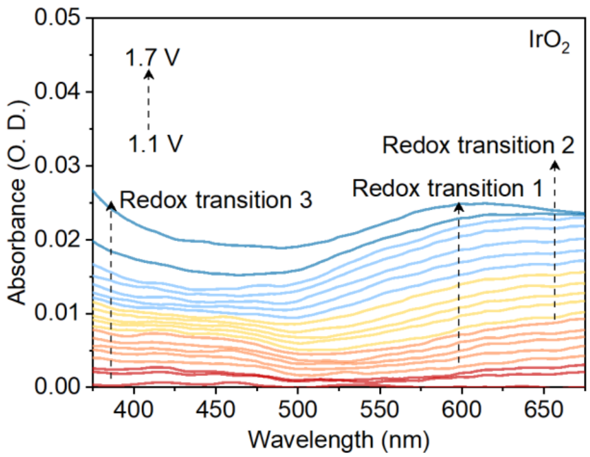

Supplementary Figure 26. In situ ultraviolet-visible spectroscopy (UV-vis) absorption spectra of IrO_2.

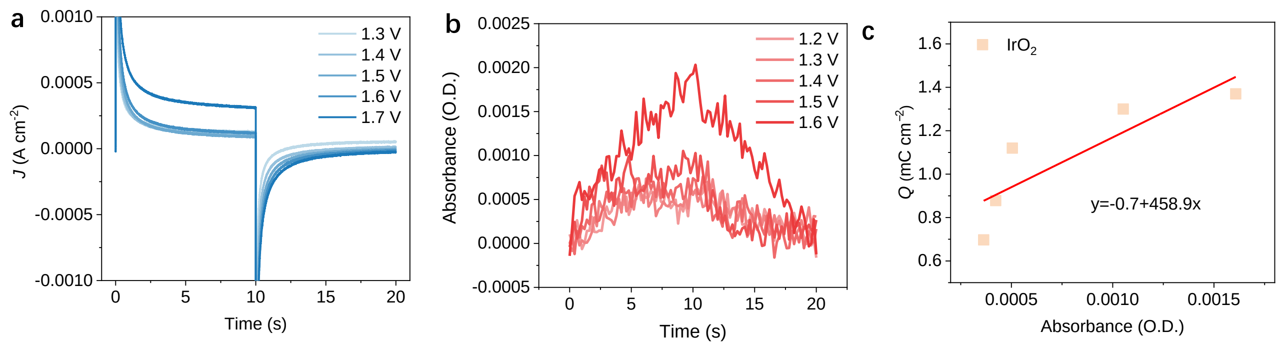

Supplementary Figure 27. a, Pulse current relationship, **b,** pulse absorbance relationship and **c,** charge absorption relationship in time resolved UV-vis absorption spectra of IrO_2.

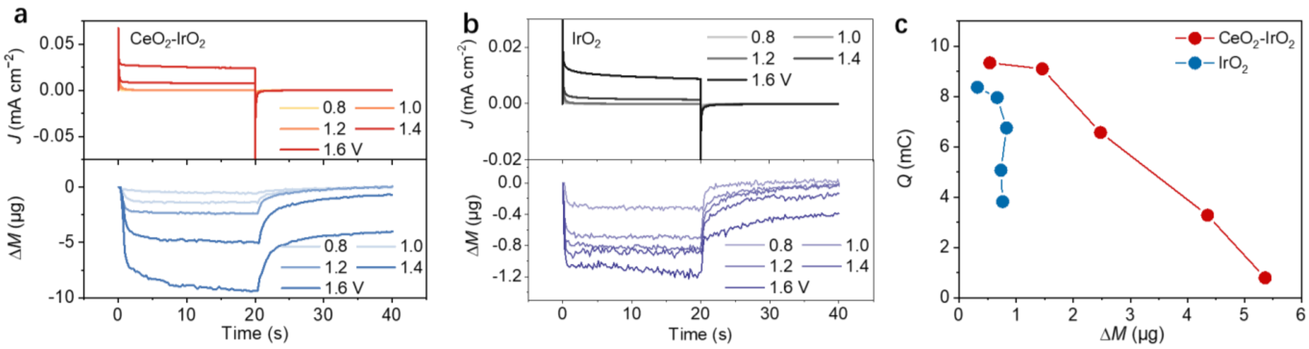

Supplementary Figure 28. a, b, EQCM measurements showing the pulsed electrochemical response and corresponding mass change of CeO_2-IrO_2. **c,** Correlation between charge and mass response for CeO_2-IrO_2 and IrO_2.

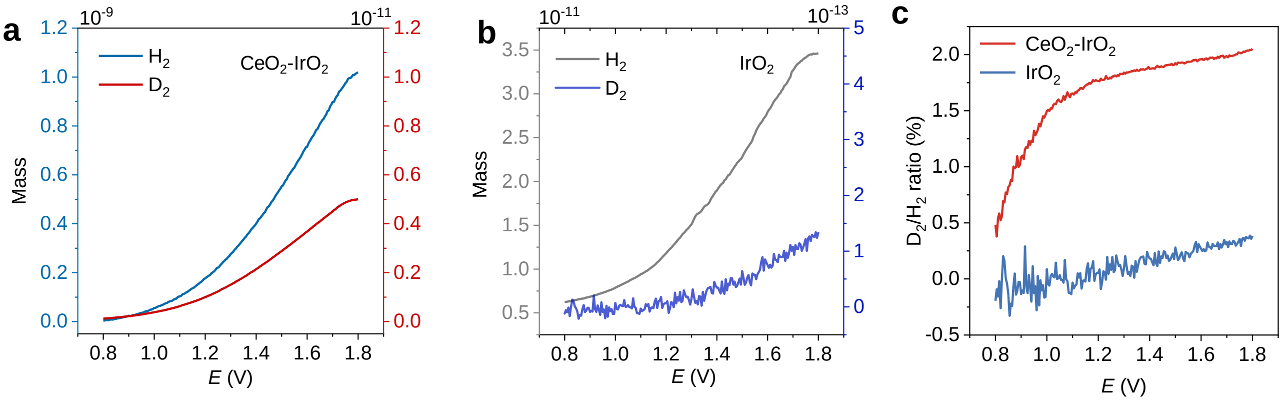

Supplementary Figure 29. a, b, The relationship between H_2 and D_2 of CeO_2-IrO_2 and IrO_2 with respect to the potential. **c,** D_2/H_2 ratio versus potential for CeO_2-IrO_2 and IrO_2 under MEA conditions.

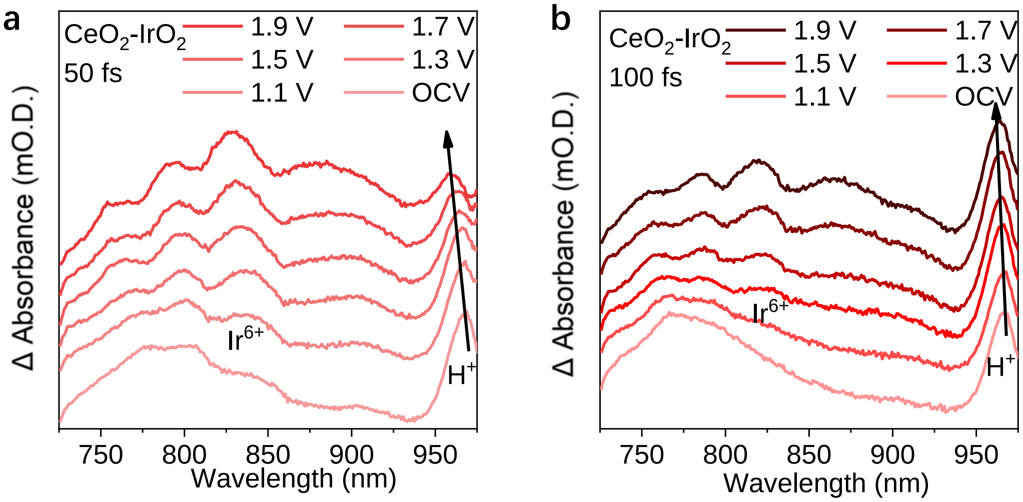

Supplementary Figure 30. a, b, TAS spectra of CeO_2-IrO_2 as a function of voltage at 50 and 100 *fs*.

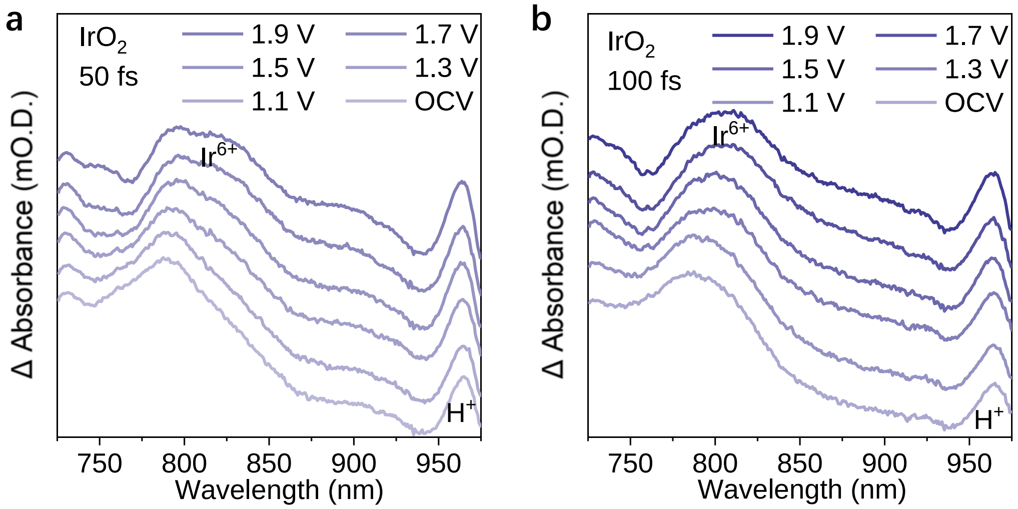

Supplementary Figure 31. a, b, TAS spectra of IrO_2 as a function of voltage at 50 and 100 *fs*.

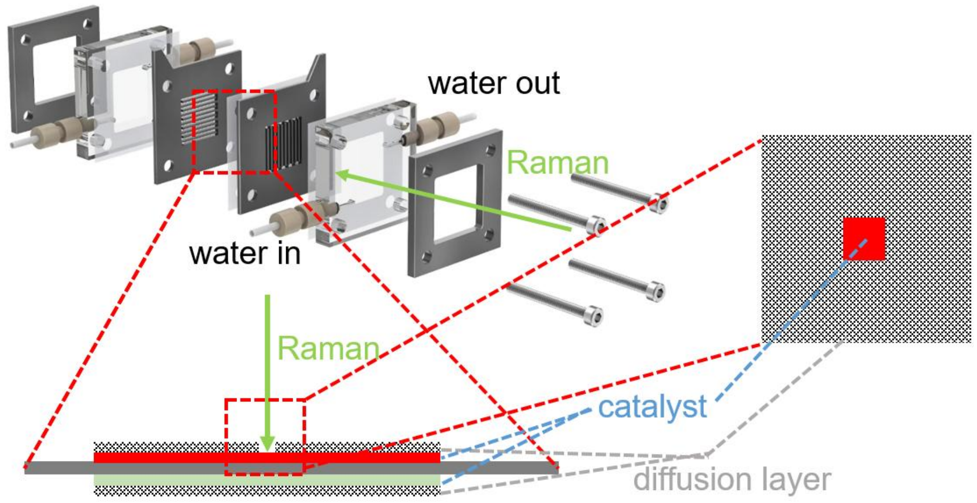

Supplementary Figure 32. Raman device for membrane electrode working condition monitoring.

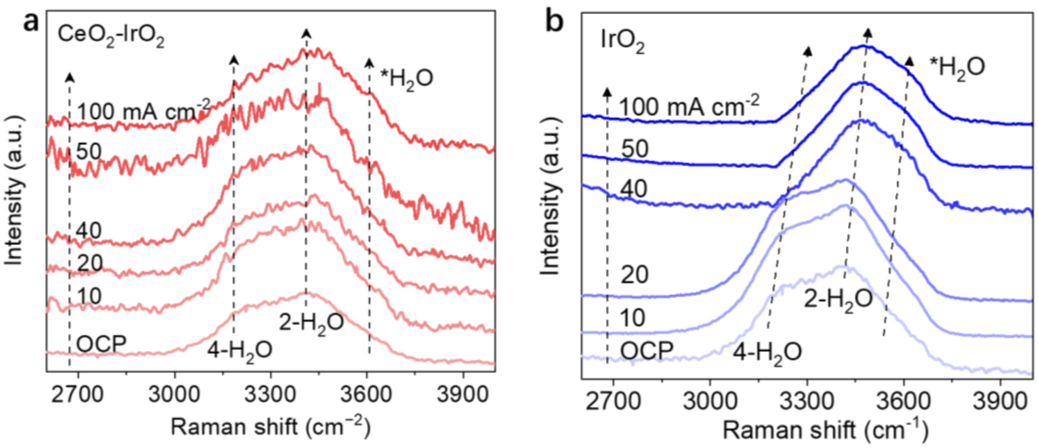

Supplementary Figure 33. a, b, Raman spectra of the membrane electrode working conditions of CeO_2-IrO_2 and IrO_2.

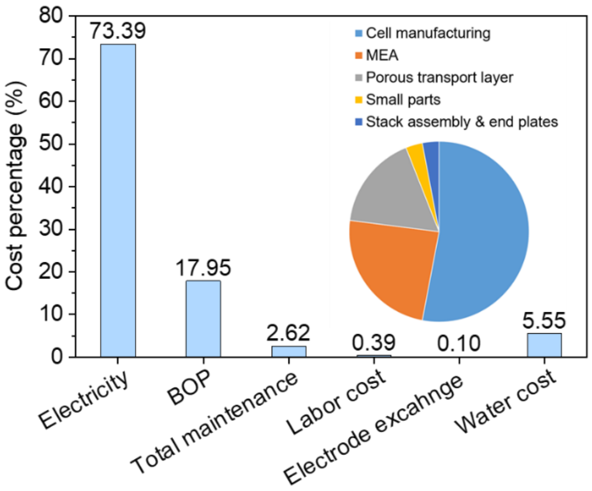

Supplementary Figure 34. Cost distribution of PEMWE for hydrogen production.

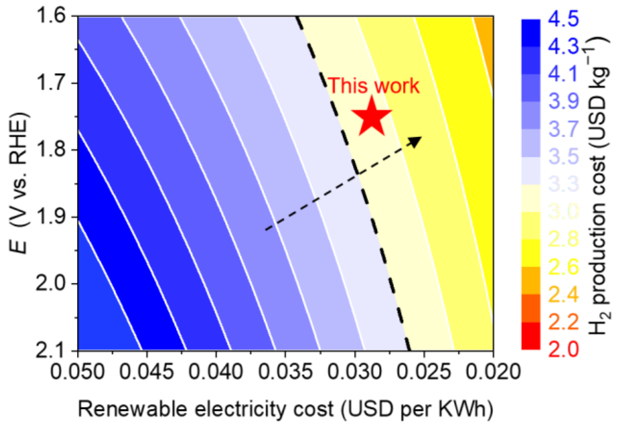

Supplementary Figure 35. The relationship between electricity price, voltage and hydrogen production cost of TEA.

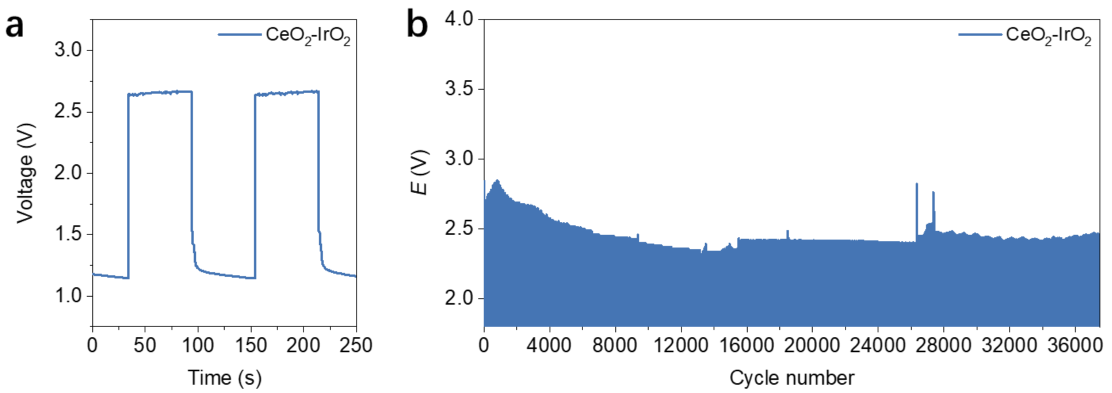

Supplementary Figure 36. a, Single-cycle start-stop test of CeO_2-IrO_2. **b,** Long-term start-stop durability test of CeO_2-IrO_2.

Supplementary References

1. Sun, X. et al. Corrosion-resistant NiFe anode towards kilowatt-scale alkaline seawater electrolysis. *Nat. Commun.* **15**, 10351 (2024).
2. Tackett, B. M., Raciti, D., Brady, N. W., Ritzert, N. L., T. Moffat, P. Potentiometric rotating ring disk electrode study of interfacial pH during CO_2 reduction and H_2 generation in neutral and weakly acidic media. *J. Phys. Chem. C* **126**, 7456-7467 (2022).
3. Chen, W., Liao, L. W., Cai, J., Chen, Y.-X. U. Stimming, Unraveling complex electrode processes by differential electrochemical mass spectrometry and the rotating ring-disk electrode technique. *J. Phys. Chem. C* **123**, 29630-29637 (2019).
4. Kucernak, A. R., Wang, H., Lin, X. Avoid using phosphate buffered saline (PBS) as an electrolyte for accurate OER studies. *ACS Energy Lett.* **9**, 3939-3946 (2024).
5. Yang, C. et al. Revealing pH-dependent activities and surface instabilities for Ni-based electrocatalysts during the oxygen evolution reaction. *ACS Energy Lett.* **3**, 2884-2890 (2018).
6. De Groot, M. T., Merkx, M., Wonders, A. H., Koper, M. T. M. Electrochemical reduction of NO by hemin adsorbed at pyrolitic graphite. *J. Am. Chem. Soc.* **127**, 7579-7586 (2005).
7. Goyal, A., Marcandalli, G., Mints, V. A., M. Koper, T. M. Competition between CO_2 reduction and hydrogen evolution on a gold electrode under well-defined mass transport conditions. *J. Am. Chem. Soc.* **142**, 4154-4161 (2020).
8. Bollella, P., Melman, A., Katz, E. Electrochemically generated interfacial pH change: application to signal-triggered molecule release. *ChemElectroChem* **7**, 3386-3403 (2020).
9. Zhang, F., Co, A. C. Direct evidence of local pH change and the role of alkali cation during CO_2 electroreduction in aqueous media. *Angew. Chem. Int. Ed.* **59**, 1674-1681 (2020).
10. Hasché, F., Oezaslan, M., Strasser, P. T.-P. Fellinger, Electrocatalytic hydrogen peroxide formation on mesoporous non-metal nitrogen-doped carbon catalyst. *J. Energy Chem.* **25**, 251-257 (2016).

11 VandeVondele, J. et al. Quickstep: Fast and accurate density functional calculations using a mixed Gaussian and plane waves approach. *Comput. Phys. Commun.* **167**, 103-128 (2005).

12 Adamo, C. & Barone, V. Toward reliable density functional methods without adjustable parameters: The PBE0 model. *J. Chem. Phys.* **110**, 6158-6170 (1999).

13 Lu, T. A comprehensive electron wavefunction analysis toolbox for chemists, Multiwfn. *J. Chem. Phys.* **161**, 082503 (2024).

14. Kresse, G., Furthmüller, J. Efficient iterative schemes for ab initio total-energy calculations using a plane-wave basis set. *Phys. Rev. B* **54**, 11169-11186 (1996).
15. Kresse, G., Joubert, D. From ultrasoft pseudopotentials to the projector augmented-wave method. *Phys. Rev. B* **59**, 1758-1775 (1999).
16. Perdew, J. P., Burke, K., Ernzerhof, M. Generalized gradient approximation made simple. *Phys. Rev. Lett.* **77**, 3865-3868 (1996).
17. Hu, Y. et al. Understanding the sulphur-oxygen exchange process of metal sulphides prior to oxygen evolution reaction. *Nat. Commun.* **14**, 1949 (2023).
18. Lin, C. et al. In-situ reconstructed Ru atom array on α-MnO_2 with enhanced performance for acidic water oxidation. *Nat. Catal.* **4**, 1012-1023 (2021).
19. Zhao, H. et al. Stabilizing lattice oxygen through Mn doping in $NiCo_2O_{4-\delta}$ spinel electrocatalysts for efficient and durable acid oxygen evolution. *Angew. Chem. Int. Ed.* **63**, e202402171 (2024).
20. Zi, S. et al. Surface cladding engineering via oxygen sulfur distribution for stable electrocatalytic oxygen production. *Angew. Chem. Int. Ed.* **64**, e202413348 (2025).
21. Guo, L. et al. Ceria-optimized oxygen-species exchange in hierarchical bimetallic hydroxide for electrocatalytic water oxidation. *Adv. Mater.* **36**, 2406682 (2024).
22. Gao, G. et al. Topological rearrangement-derived edge-sharing [MO_6] motifs on perovskite oxide for optimizing O-O bonding in water oxidation. *Chem. Eng. J.* **502**, 157869 (2024).
23. Dou, Q., Liu, L., Yang, B., Lang, J., Yan, X. Silica-grafted ionic liquids for revealing the respective charging behaviors of cations and anions in supercapacitors. *Nat. Commun.* **8**, 2188 (2017).
25. Li, Z. et al. Alcohols electrooxidation coupled with H_2 production at high current densities promoted by a cooperative catalyst. *Nat. Commun.* **13**, 147 (2022).
26. Liu, T. et al. In situ quantification of interphasial chemistry in Li-ion battery. *Nat. Nanotechnol.* **14**, 50-56 (2019).
27. Bendadesse, E. et al. Deciphering the double-layer structure and dynamics on a model Li_xMoO_3 interface by advanced electrogravimetric analysis. *ACS Nano* **16**, 14907-14917 (2022).
28. Ji, Y. et al. From bulk to interface: electrochemical phenomena and mechanism studies in batteries via electrochemical quartz crystal microbalance. *Chem. Soc. Rev.* **50**, 10743-10763 (2021).

29. Platek-Mielczarek, A., Frackowiak, E., Fic, K. Specific carbon/iodide interactions in electrochemical capacitors monitored by EQCM technique. *Energy Environ. Sci.* **14**, 2381-2393 (2021).
30. Xia, Y. et al. A cost-effective alkaline polysulfide-air redox flow battery enabled by a dual-membrane cell architecture. *Nat. Commun.* **13**, 2388 (2022).
31. Xu, Z. et al. Chemical upcycling of polyethylene, polypropylene, and mixtures to high-value surfactants. *Science* **381**, 666-671 (2023).
32. Li, J. et al. Rechargeable biomass battery for electricity storage/generation and concurrent valuable chemicals production. *Angew. Chem. Int. Ed.* **62**, e202304852 (2023).
33. Ren, J.-T., Chen, L., Wang, H.-Y., Tian, W.-W., Yuan, Z.-Y. Water electrolysis for hydrogen production: from hybrid systems to self-powered/catalyzed devices. *Energy Environ. Sci.* **17**, 49-113 (2024).
34. Chen, D., Li, W., Liu, J., Sun, L. Bio-inspired proton relay for promoting continuous 5-hydroxymethylfurfural electrooxidation in a flowing system. *Energy Environ. Sci.* **18**, 3120-3128 (2025).
